# Essential roles of lytic transglycosylases in l,d-transpeptidase-mediated peptidoglycan cross-linking in *Escherichia coli*

**DOI:** 10.1128/spectrum.01017-26

**Published:** 2026-09-21

**Authors:** Constantin Anoyatis-Pelé, Mathilde Benadjaoud, Michel Arthur, Jean‑Emmanuel Hugonnet

**Affiliations:** 1 INSERM ERL 1336, UMRS 8228, Sorbonne Université-ENS-PSL-CNRS, Paris, France; Texas A&M University, College Station, Texas, USA; Cornell University, Ithaca, New York, USA

**Keywords:** peptidoglycan, lytic transglycosylases, L,D-transpeptidases, *Escherichia coli*, autolysins, endopeptidases

## Abstract

Bacteria resist the turgor pressure of the cytoplasm thanks to peptidoglycan, a mesh-like macromolecule that completely surrounds the cytoplasmic membrane and consists of glycan chains cross-linked to each other by short peptides. Protecting the cell from swelling and lysing during the entire cell cycle, while enabling cell expansion and division, requires the coordinated action of enzymes that form (glycosyltransferases and transpeptidases) and cleave (lytic transglycosylases, endopeptidases, and amidases) peptidoglycan chemical bonds. In *Escherichia coli*, cleavage of glycosidic bonds is potentially performed by eight lytic transglycosylases with partially redundant functions. Here, we explore the roles of these enzymes in bacteria that overproduce the penicillin-insensitive L,D-transpeptidase YcbB (LdtD), leading to the bypass of conventional D,D-transpeptidases belonging to the penicillin-binding protein family and to resistance to β-lactam antibiotics. Construction of lytic transglycosylase-deficient mutants and cross-complementation with other members of the autolysin family identified combinations of enzymes that are essential either for the expression of YcbB-mediated β-lactam resistance or for the prevention of toxic effects resulting from YcbB overproduction in the absence of β-lactams. We show that, among the eight lytic transglycosylases, MltG and MltD have both essential and non-redundant roles in these conditions and that endopeptidase MepK can compensate for the absence of MltG. We propose that MltG and MltD specifically alleviate defects in peptidoglycan cross-linking by eliminating toxic peptidoglycan intermediates that potentially accumulate in the inner and outer side of the peptidoglycan layer of bacterial cells overproducing YcbB, respectively.

**IMPORTANCE:**

The role of lytic transglycosylases has been extensively studied in bacteria that rely on conventional D,D-transpeptidases (PBPs) for PG cross-linking. In contrast, little is known about the contribution of these enzymes in the PG metabolism that results after the bypass of PBPs by L,D-transpeptidase YcbB (aka LdtD). Here, we show that lytic transglycosylases MltG and MltD have specific and non-redundant roles in YcbB-mediated cross-linking. We also show that the absence of these autolysins can be partially compensated by the overproduction of endopeptidase MepK. MltG fulfilled a dual function involving both the production of the substrate of YcbB and the elimination of aberrant toxic peptidoglycan intermediates. The non-redundant roles of MltG and MltD could be accounted for by the elimination of these toxic intermediates from the inner and outer side of the PG layer, respectively. These results expand the repertoire of the physiological roles of lytic transglycosylases in PG metabolism.

## INTRODUCTION

Peptidoglycan (PG) is an essential bacterial macromolecule consisting of glycan chains linked to each other by short peptides. This net-like structure counteracts the turgor pressure of the cytoplasm and determines bacterial cell shape (1). PG assembly involves glycosyltransferases that polymerize glycan chains and transpeptidases that form peptide cross-links connecting adjacent glycan chains. In most bacterial species, PG cross-links are predominantly or exclusively formed by transpeptidases of the D,D specificity that cleave the D‐Ala^4^‐D‐Ala^5^ bond of an acyl donor stem and link D‐Ala^4^ to diaminopimelate (DAP^3^) of an acceptor stem peptide, thereby forming 4→3 cross-links (2). These enzymes are members of the penicillin-binding protein (PBP) family and are the essential targets of β-lactam antibiotics. The remaining cross-links are formed by transpeptidases of the L,D specificity (L,D-transpeptidases). These enzymes catalyze the formation of an amide bond between DAP residues at the third position of donor and acceptor stems (3→3 cross-links) (3, 4). In *E. coli*, L,D-transpeptidases are non-essential enzymes that have a minor (≈10%) contribution to PG cross-linking. However, the overproduction of L,D-transpeptidase YcbB (aka LdtD) results in the partial (≈40%) replacement of 4→3 by 3→3 cross-links (5). In combination with the overproduction of the alarmone guanosine tetraphosphate and pentaphosphate [(p)ppGpp] by the plasmid-encoded RelA’ enzyme, overproduction of YcbB leads to β-lactam resistance due to bypass of PBPs by YcbB. This bypass is complete in bacteria grown in the presence of β-lactams as experimentally established by the full replacement of 4→3 by 3→3 cross-links (6). The essential role of (p)ppGpp in β-lactam resistance stems from the role of the alarmone in controlling the transcription of specific ribosomal RNA operons (5, 7).

**Fig 1:**
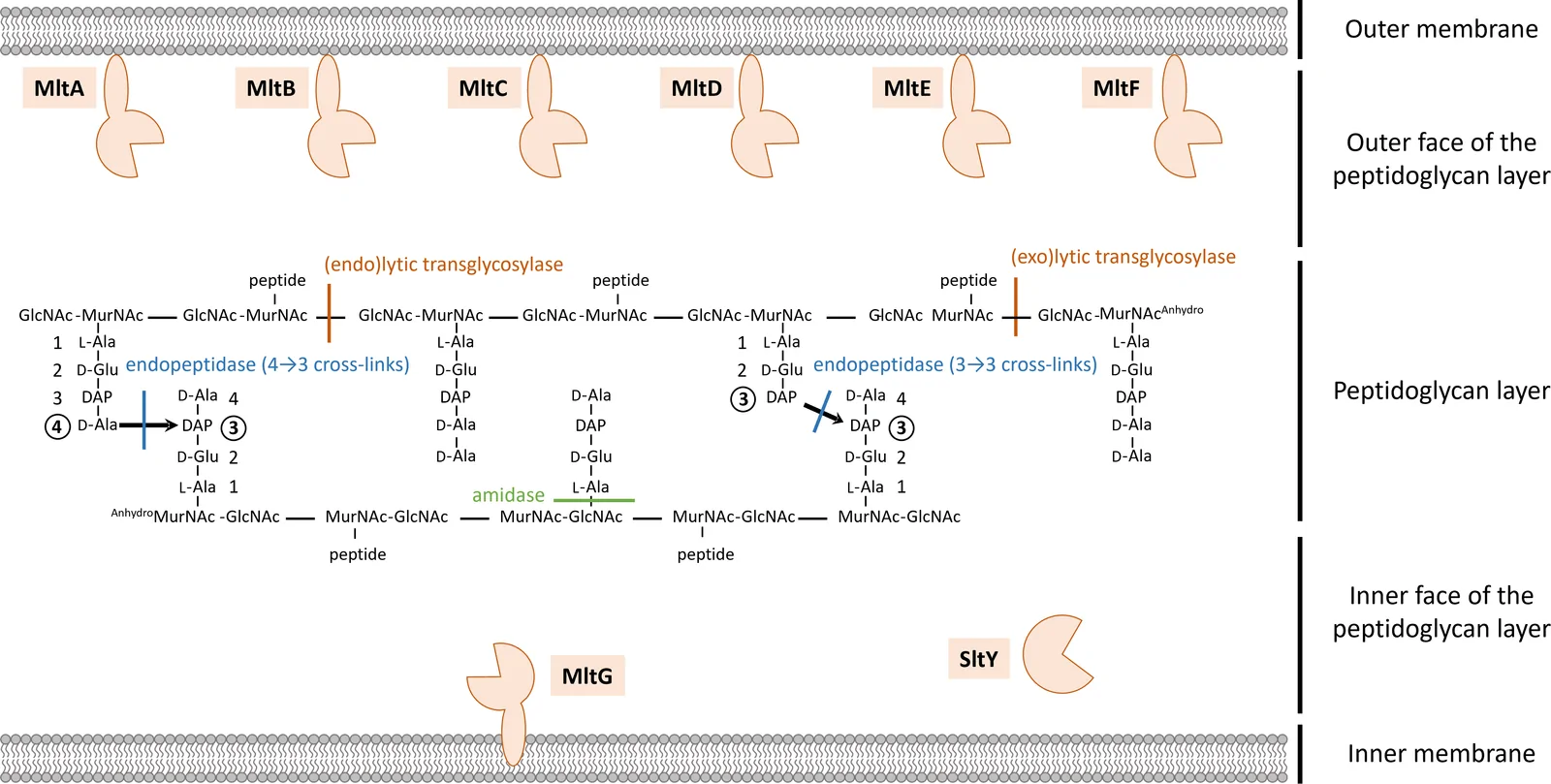
Chemical bonds cleaved by the autolysins and localization of the lytic transglycosylases. *E. coli* autolysins comprise three families of enzymes. The lytic transglycosylases (in orange) cleave MurNAc-GlcNAc bonds forming anhydro-MurNAc residues. They can act on internal (endo) or terminal (exo) residues. Endopeptidases (in blue) cleave either 4→3 and/or 3→3 cross-links. Amidases (in green) hydrolyze the MurNAc-L-Ala bond. The eight active lytic transglycosylases of *E. coli* are depicted according to their localization, anchored to the outer membrane *via* a palmitoylated cysteine residue (MltA, B, C, D, E, and F), soluble in the periplasm (SltY), or entwined in the inner membrane *via* a hydrophobic domain (MltG). DAP, diaminopimelic acid; GlcNAc, *N*-acetylglucosamine; MurNAc, *N*-acetylmuramic acid.

PG metabolism also involves a variety of enzymes that potentially cleave all glycosidic and amide bonds in the PG polymer (Fig. 1) (8, 9). These enzymes are referred to as autolysins because their unregulated activity can potentially lead to bacterial cell lysis (10, 11). The eight-member family of lytic transglycosylases catalyze a non-hydrolytic cleavage of the β-1,4 MurNAc-GlcNAc glycosidic bond that leads to the formation of an intramolecular bond connecting positions 1 and 6 of MurNAc residues (12). The resulting anhydro-MurNAc residue is the hall mark of lytic transglycosylases. These enzymes have an endolytic activity, cleaving β-1,4 bonds internal to glycan chains, or an exolytic activity, processively cleaving glycan chains from their reducing end (13). The endolytic and exolytic activities of lytic transglycosylases have numerous functions including the elimination of toxic PG polymers, PG turnover, and PG recycling (14, 15). In addition, lytic transglycosylases act as terminases to cleave nascent glycan chains, thereby terminating glycan chain elongation (16–18). Six of the eight lytic transglycosylases (MltA, B, C, D, E, F) are lipoproteins anchored into the inner leaflet of the outer membrane (Fig. 1; Fig. S1). Thus, their catalytic domains face the outer side of the PG layer. SltY is a soluble enzyme. MltG is anchored into the inner membrane by its hydrophobic N-terminal extremity. *E. coli* also produces RlpA, a homolog of the lytic transglycosylase RlpA of *Pseudomonas aeruginosa*. Although this enzyme has been shown to be an active lytic transglycosylase in *P. aeruginosa*, RlpA produced by *E. coli* is inactive. This could be due to a substitution of the catalytic glutamate residue by a serine (19) (Fig. S1).

The endopeptidases form a heterogeneous family of eight proteins that cleave the amide bonds formed either or both by transpeptidases of the D,D and L,D specificities. Endopeptidases belonging to the PBP family (PBP4, PBP7, and AmpH) only cleave 4→3 cross-links (20–22). The remaining endopeptidases (MepM, MepK, MepS, MepA, and MepH) cleave both 4→3 and 3→3 cross-links although MepK is more effective in cleaving 3→3 than 4→3 cross-links (23–26). The endopeptidases have been shown to be essential for the one-at-a-time incorporation of newly synthesized glycan strands into the expanding side wall (27).

Autolysins include a third family of enzymes, referred to as amidases, which cleave the MurNAc-L-Ala amide bond. These enzymes are involved in the separation of daughter cells at the end of the cell cycle (28). This function is achieved by the controlled hydrolysis of MurNAc-L-Ala amide bonds in newly synthesized septal PG (29). Amidases are also involved in PG recycling (30).

It was previously shown that the presence of 4→3 and 3→3 cross-links affects the catalytic activity of lytic transglycosylases (31). However, it is not known whether certain lytic transglycosylases specifically participate in either mode of PG cross-linking. Here, we evaluated the role of lytic transglycosylases in the growth and β-lactam resistance of bacteria that partially or exclusively rely on the L,D-transpeptidase activity of YcbB for PG cross-linking. We showed that two out of the eight lytic transglycosylases, MltD and MltG, are specifically essential for the bypass of conventional D,D-transpeptidases belonging to the PBP family by YcbB. We then determined by high-resolution mass spectrometry the cleavage specificity of MltG and MltD to identify the substrates and products of these enzymes that might account for their essential role in resistance and growth of YcbB-overproducing bacteria. We also evaluated whether the overproduction of autolysins belonging to the endopeptidase family could compensate for the absence of MltG or MltD. Based on these analyses, we propose that MltG and MltD have dual roles in PG metabolism that involve both the generation of substrates for YcbB and the elimination of aberrant toxic PG intermediates that potentially accumulate in the inner and outer face of the PG layer.

## RESULTS

### MltD and MltG are essential for YcbB-mediated β-lactam resistance

**Fig 2:**
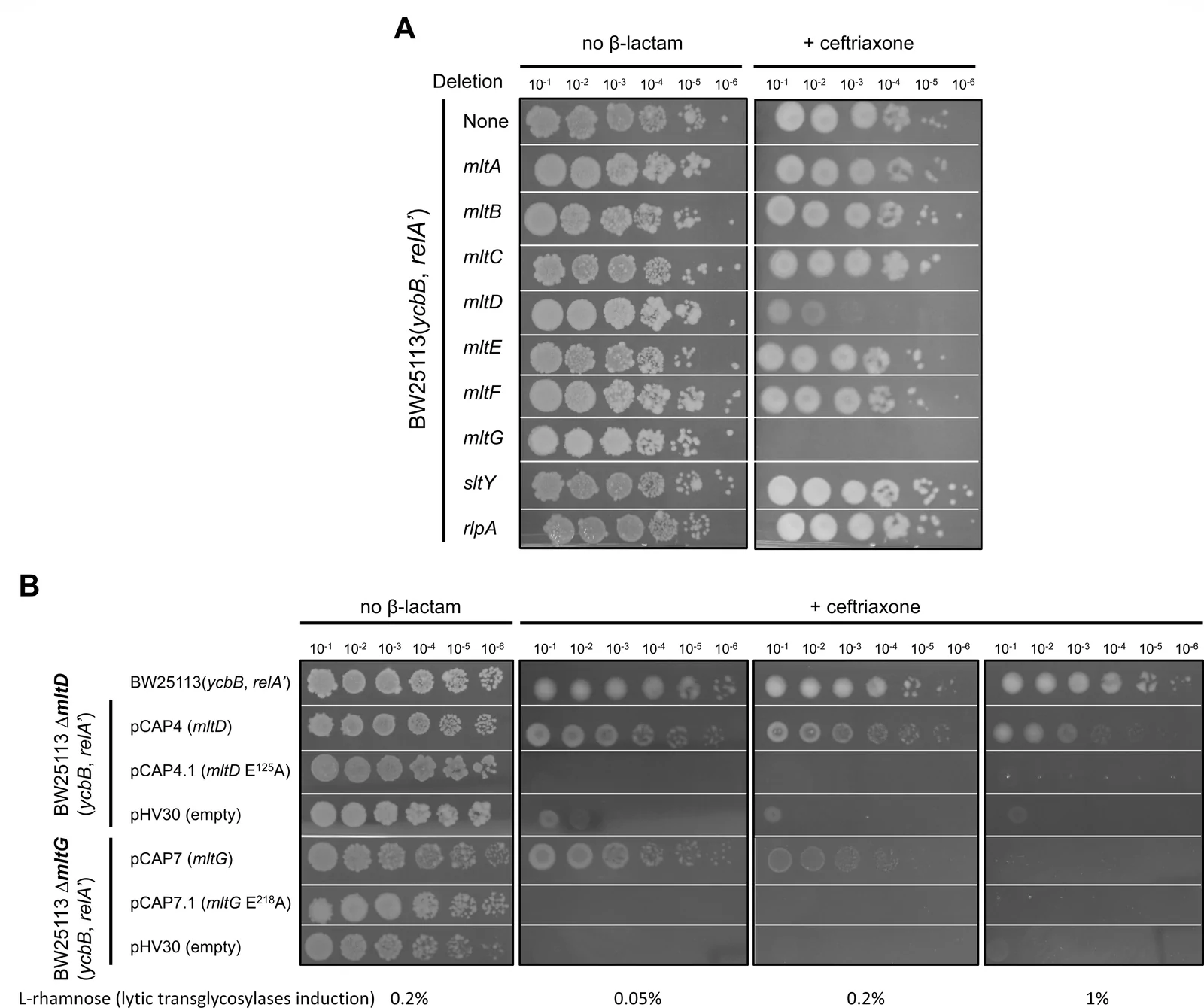
Essential lytic transglycosylases in YcbB-mediated β-lactam resistance. (**A**) The plating efficiency of BW25113 (*ycbB*, *relA’*) or its derivatives harboring individual deletions of each one of the eight genes encoding active lytic transglycosylases, or *rlpA*, was tested in BHI agar plates supplemented with 40 µM IPTG, 1% L-arabinose, and 8 µg/mL ceftriaxone (+ ceftriaxone) or in the absence of the drug (- β-lactam). *E. coli* BW25113 (*ycbB*, *relA’*) harbors (i) plasmid pKT2 carrying an isopropyl-β-D-thiogalactopyranoside (IPTG) inducible copy of *ycbB*, (ii) plasmid pKT8 carrying an L-arabinose-inducible copy of the *relA’* allele of the (p)ppGpp synthetase gene, and (iii) a deletion of the chromosomal copy of the *relA* gene (26). Induction of *ycbB* by IPTG and of *relA’* by L-arabinose enables bypass of the cross-linking activity of PBPs by that of YcbB resulting in ceftriaxone resistance because YcbB is not inhibited by β-lactams of the penam or cephem classes. (**B**) Complementation of the *mltD* or *mltG* deletions by derivatives of the pHV30 plasmid enabling L-rhamnose-inducible production of the functional MltD or MltG lytic transglycosylases or their catalytically inactive derivatives MltD E^125^A or MltG E^218^A. The concentration of L-rhamnose (%) for induction of lytic transglycosylase genes varied as indicated at the bottom of the panels. As a control, strains were transformed with the pHV30 vector (empty). Plates shown correspond to one of three independent experiments (*n* = 3).

The eight lytic transglycosylases of *E. coli* (Fig. 1; Fig. S1) were previously shown to have redundant functions because individual deletions of the corresponding genes lead to viable mutants (28). Our first objective was to determine whether any of these proteins were required in conditions in which the D,D-transpeptidase activity of the PBPs is replaced by the L,D-transpeptidase activity of YcbB. The eight lytic transglycosylase genes were, thus, individually deleted from the chromosome of *E. coli* BW25113 (*ycbB*, *relA*’), which is resistant to ceftriaxone due to the bypass of PBPs by YcbB and the production of (p)ppGpp by RelA’ (6, 26). The deletion of *mltG* or *mltD*, abolished or impaired the expression of YcbB-mediated ceftriaxone resistance, respectively, whereas the deletion of the six-remaining lytic transglycosylase genes, or the gene encoding RlpA, had no effect (Fig. 2A). The deletion of *mltD* or *mltG* was found to be complemented by the wild-type genes but not by the genes encoding the proteins with an alanine substitution of the catalytic glutamate residue (E^125^ for MltD and E^218^ for MltG; Fig. 2B). Thus, the lytic transglycosylase activity of MltD and MltG was crucial for YcbB-mediated β-lactam resistance.

### MltD and MltG have non-redundant and essential functions in β-lactam resistance

**Fig 3:**
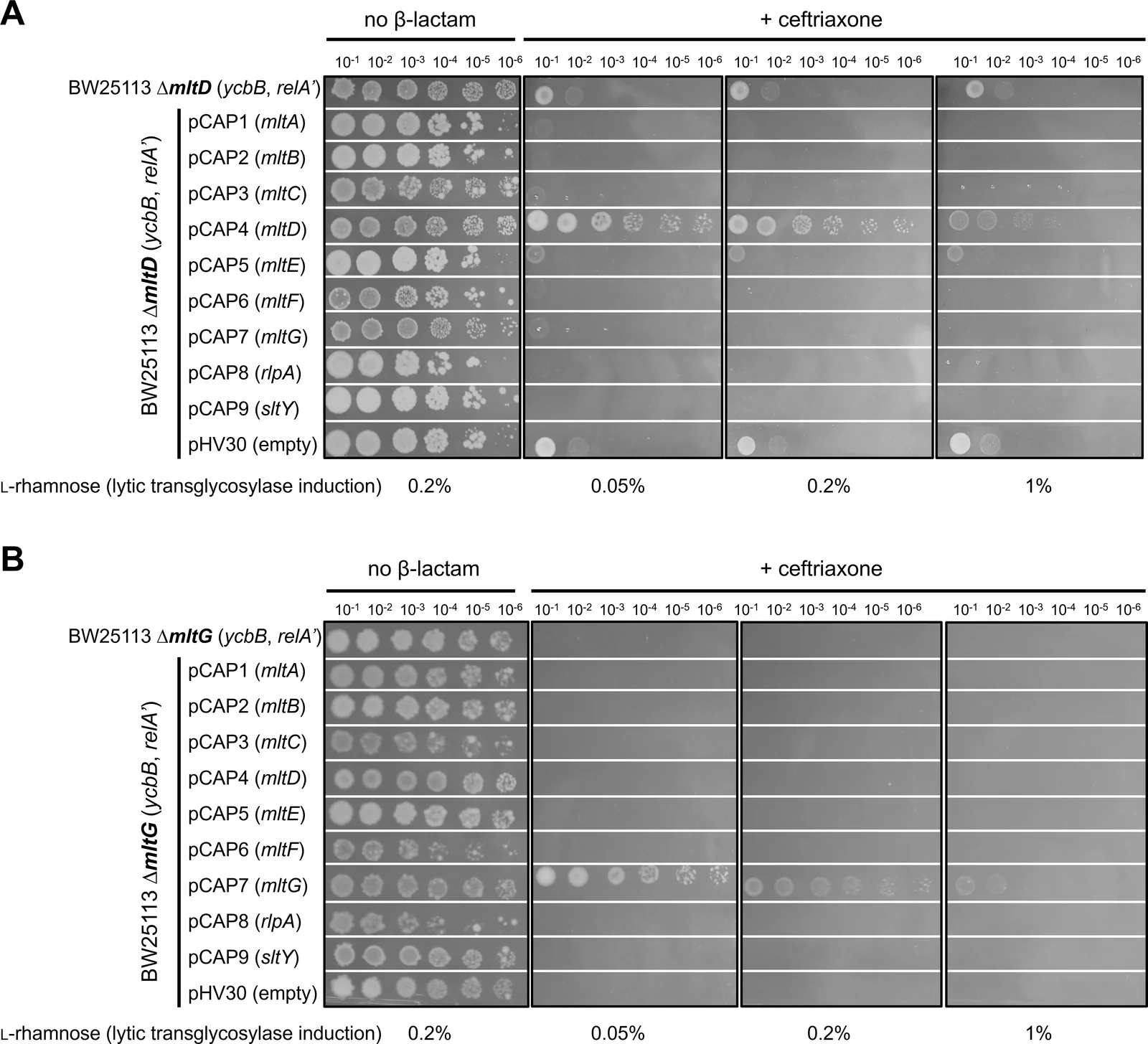
Complementation assay of the *mltD* and *mltG* mutants by the other lytic transglycosylases. (**A**) The plating efficiency of BW25113 (*ycbB, relA’*) ∆*mltD* or its derivatives harboring plasmids enabling L-rhamnose-inducible expression of the genes encoding lytic transglycosylases was tested in BHI agar plates supplemented with 40 µM IPTG, 1% L-arabinose, 8 µg/mL ceftriaxone (+ ceftriaxone), or in the absence of the drug (- β-lactam). The concentration of L-rhamnose (%) for induction of lytic transglycosylase genes varied as indicated at the bottom of the panels. (**B**) The same assay as described in (**A**) was also performed for the BW25113 ∆*mltG* (*ycbB, relA’*) strain. As a control, strains were transformed with the pHV30 vector (empty). Plates shown correspond to one of three independent experiments (*n* = 3).

We then evaluated heterologous complementation of the *mltD* and *mltG* deletions by each one of the seven other lytic transglycosylase genes (Fig. 3A and B). The *mltD* and *mltG* deletions were only complemented by the expression of the corresponding genes; therefore, none of the heterologous complementations restored resistance. Thus, MltD and MltG each have non-redundant roles in YcbB-mediated β-lactam resistance.

### MltD and MltG cleave glycan strands more effectively than cross-linked PG

**Fig 4:**
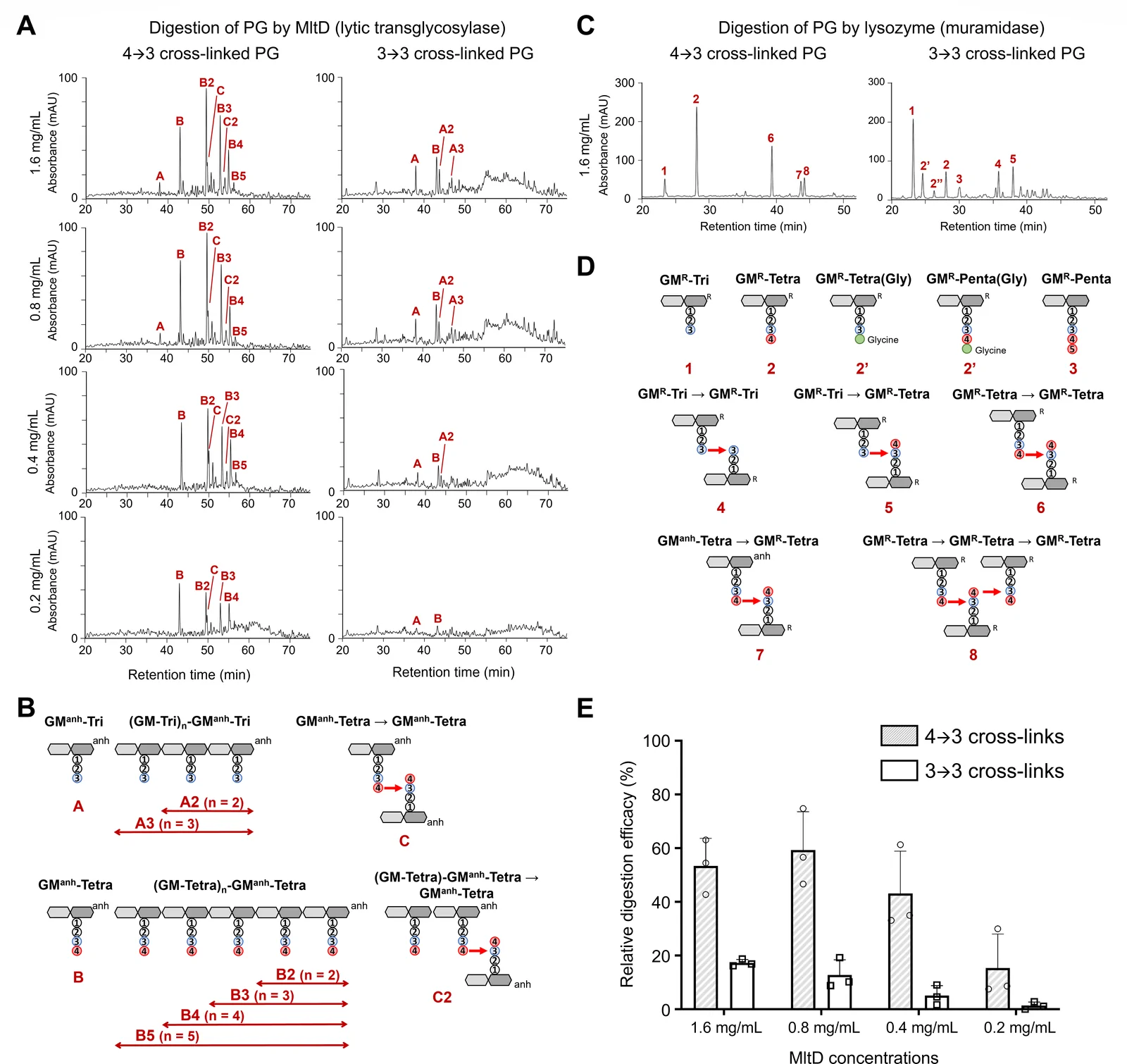
Digestion of 4→3 and 3→3 cross-linked sacculi by MltD. (**A**) *rp*HPLC muropeptide profiles obtained by the digestion of the same amount of 4→3 or 3→3-cross-linked sacculi (columns) by various concentrations of MltD (rows). (**B**) Structure of the main muropeptides generated by digestion of the sacculi by MltD, as determined by mass spectrometry. (**C**) *rp*HPLC muropeptide profiles obtained by full digestion of 4→3 and 3→3 cross-linked sacculi by lysozyme (same PG amount as in [**A**]). (**D**) Structure of the muropeptides generated by digestion of sacculi by lysozyme, as determined by mass spectrometry. (**E**) Efficacy of digestion of 4→3 vs 3→3 cross-linked PG by MltD. The relative proportion (%) of MltD- vs lysozyme-generated muropeptides was determined by calculating the ratio of the sums of the peak areas (peaks A, B, C, A2, A3, B1, B2, B3, and B4) for MltD vs those (1, 2, 2’, 2’’, 3, 4, 5, 6, and 7) for lysozyme. Data are shown as means ± standard deviation (s.d.) (only shown as mean + s.d. for clarity) from three independent experiments. Light and dark gray hexagons represent *N*-acetylglucosamine (GlcNAc) and *N*-acetylmuramic acid (MurNAc) residues, respectively. Anh and R represent anhydro and reduced ends, respectively. Amino acid residues of peptide stems are numbered (1, L-Ala; 2, D-*iso*-glutamate [D-iGlu]; 3, diaminopimelic acid [DAP]; 4 and 5, D-Ala). Tri, Tetra, and Penta indicate stem peptides comprising 3, 4, and 5 amino acid residues. mAU, milliabsorbance units, *λ* = 205 nm. Raw data used to generate this figure are provided in Table S3.

**Fig 5:**
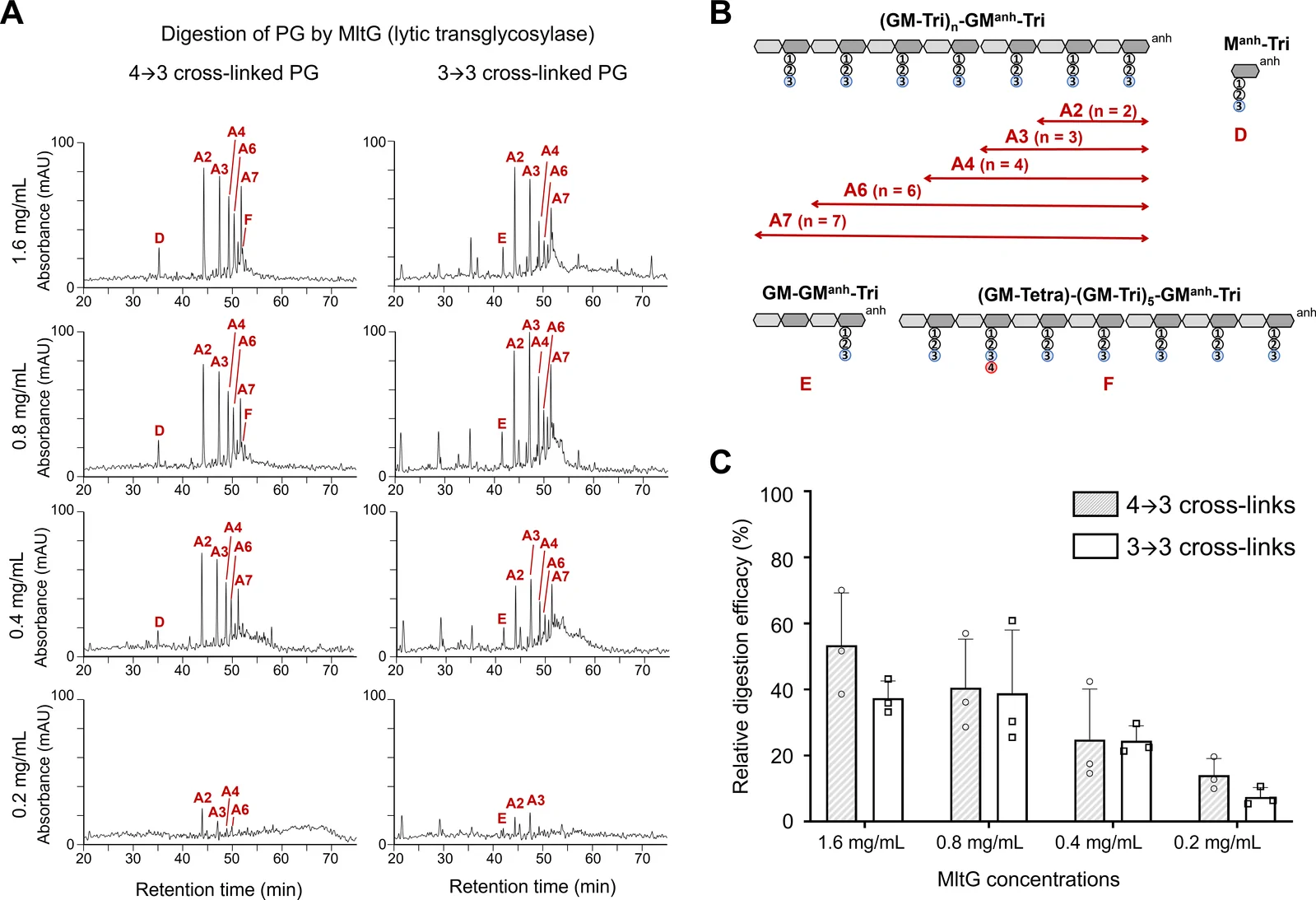
Digestion of 4→3 and 3→3 cross-linked sacculi by MltG. (**A**) *rp*HPLC muropeptide profiles obtained by the digestion of the same amount of 4→3 or 3→3 cross-linked sacculi (columns) by various concentrations of MltG (rows). (**B**) Structure of the main muropeptides generated by digestion of the sacculi by MltG, as determined by mass spectrometry. (**C**) Efficacy of digestion of 4→3 vs 3→3 cross-linked PG by MltG. The relative proportion (%) of MltG- vs lysozyme-generated muropeptides was determined by calculating the ratio of the sums of the peak areas (peaks A2, A3, A4, A6, A7, D, E, and F) for MltG vs those (1, 2, 2’, 2’’, 3, 4, 5, 6, and 7) for lysozyme. Data are shown as means ± standard deviations from three independent experiments. Light and dark gray hexagons represent *N*-acetylglucosamine (GlcNAc) and *N*-acetylmuramic acid (MurNAc) residues, respectively. Anh and R represent anhydro and reduced ends, respectively. Amino acid residues of peptide stems are numbered (1, L-Ala; 2, D-*iso*-glutamate [D-iGlu]; 3, diaminopimelic acid [DAP]; 4 and 5, D-Ala). Tri, Tetra, and Penta indicate stem peptides comprising 3, 4, and 5 amino acid residues, respectively. mAU, milliabsorbance units, *λ* = 205 nm. Raw data used to generate this figure are provided in Table S4.

To assess the cleavage specificity of MltD and MltG, these enzymes were incubated with 4→3 or 3→3 cross-linked sacculi, i.e., the purified PG macromolecule (Fig. 4A and 5A). The structure of muropeptides generated by the cleavage of these sacculi by various concentrations of purified MltD and MltG was determined by *rp*HPLC and mass spectrometry (Fig. 4B and 5B). Glycosidic bonds were partially digested, leading to muropeptides containing one to five and two to seven disaccharide-peptide units for MltD and for MltG, respectively. A similar cleavage specificity was previously reported for MltG, which was shown to act on nascent glycan strands (16, 18). Thus, MtG and MltD displayed endolytic activity leading to the release of short glycan chains.

To quantitatively evaluate the extent of PG digestion by MltD, we used lysozyme to obtain a full digestion of the same amount of sacculi (Fig. 4C and D). At all tested concentrations of MltD, the sum of the areas of all *rp*HPLC-resolved muropeptide peaks was smaller for digestion of sacculi with MltD than for digestion with lysozyme (Fig. 4E). Thus, the sacculi were only partially digested by MltD. At the highest concentration of MltD that was tested, the proportion of digested PG was 54% and 17% for 4→3 and 3→3 cross-linked PG, respectively. Thus, MltD displayed a preference for 4→3 cross-linked PG. Of note, a similar preference was reported for lytic transglycosylases produced by *Vibrio cholerae* (31). For the digestion of 4→3 cross-linked PG, the proportion of muropeptides containing cross-links among all muropeptides was higher for sacculi fully digested by lysozyme than for sacculi digested by the highest concentration of MltD (41% ± 3%; peaks 6, 7, and 8 vs 8% ± 2%; peaks C and C2, respectively). Muropeptides containing cross-links were not detected in the products of digestion of 3→3 cross-linked PG by MltD. Thus, MltD preferentially cleaves uncross-linked regions of the PG, as previously reported (13, 32).

The specificity of MltG was investigated as described above for MltD (Fig. 5). MltG displayed similar activities for 4→3 and 3→3 cross-linked sacculi. Both types of sacculi were only partially digested by MltG. The muropeptides released from PG by MltG did not contain any cross-linked dimer indicating that uncross-linked PG regions are preferentially cleaved.

Together, these results indicate that MltD and MltG are both endolytic transglycosylases that display a strong preference for uncross-linked regions of the PG. This specificity indicates that both enzymes are likely to be involved in the cleavage of uncross-linked glycan strands. MltD preferentially cleaves 4→3 cross-linked PG, whereas MltG does not display this preference. The cleavage preference of MltD and MltG for uncross-linked PG indicates that these lytic transglycosylases act on glycan chains prior to their cross-linking by transpeptidases. This could account for the fact that MltD is essential in cells predominantly producing 3→3 cross-linked PG although this lytic transglycosylase does not display a preference for PG containing this type of cross-links. The specificity of MltD for uncross-linked PG indicates that this enzyme could also act in combination with endopeptidases. However, it should be acknowledged that differences in the stability and purity of our preparations of MltG and MltD might account for minor differences detected in the substrate specificity of these enzymes.

### Overproduction of endopeptidase MepK restores β-lactam resistance of the Δ*mltG* mutant

**Fig 6:**
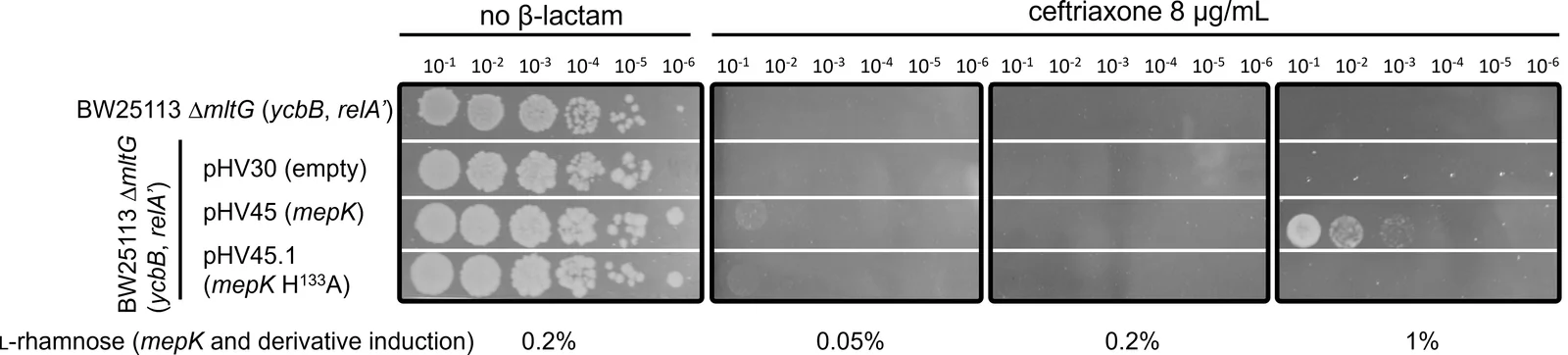
Complementation of the *mltG* deletion by the production of endopeptidase MepK for the expression of β-lactam resistance. Complementation of the *mltG* deletion was tested using derivatives of the pHV30 plasmid enabling L-rhamnose-inducible production of either functional MltG or MepK or catalytically inactive MepK H^133^A. As a control, strains were transformed with the pHV30 vector (empty). Data are representative of three independent experiments (*n* = 3).

It has recently been shown that the overproduction of lytic transglycosylase MltD restores the viability of *E. coli* strains deficient in the production of essential endopeptidases, indicating that complementation between autolysins with different cleavage specificities should be considered (27, 32). We performed the reverse complementation assay, i.e., the complementation of the *mltD* deletion by the individual overexpression of each one of the eight endopeptidase genes. The overexpression of none of these genes restored β-lactam resistance of the Δ*mltD* mutant (Fig. S2). In contrast, the overexpression of MepK restored resistance of a Δ*mltG* mutant. This partial complementation was dependent upon the endopeptidase activity of MepK because resistance was not observed for the overexpression of a gene encoding catalytically inactive MepK harboring the H^133^A substitution (Fig. 6). We also tested complementation of the Δ*mltD* and Δ*mltG* mutation by genes encoding members of the amidase enzyme family. The overproduction of none of the *E. coli* amidases (AmiA, AmiB, or AmiC) restored resistance of the Δ*mltD* and Δ*mltG* mutants (Fig. S3).

### The toxicity incurred by YcbB overproduction in a Δ*mepK* mutant is mitigated by outer membrane lipoprotein MltD

**Fig 7:**
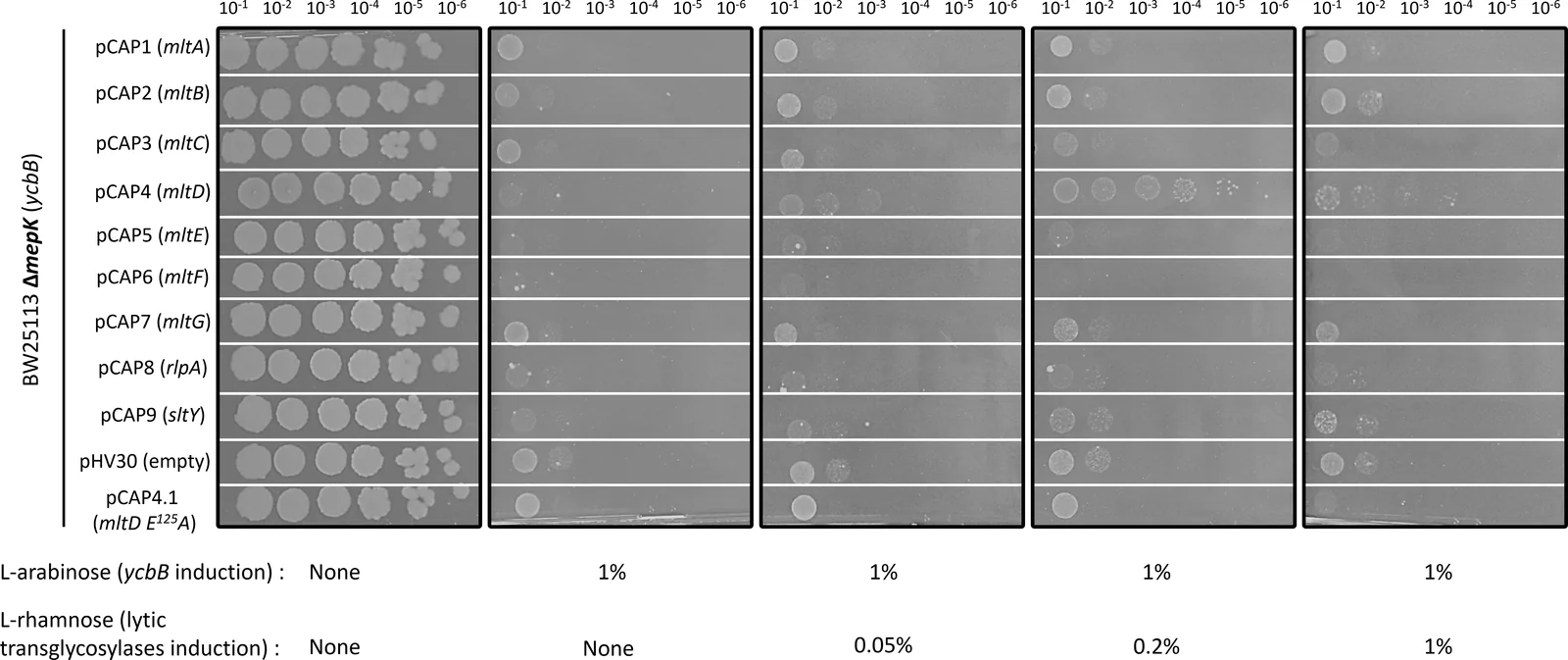
The overproduction of lytic transglycosylases for the alleviation of YcbB toxicity in a mutant deficient for the MepK endopeptidase. Derivatives of BW25113 ∆*mepK* harboring the pHV63 plasmid allowing L-arabinose-mediated expression of *ycbB* alone, or in combination with derivatives of the pHV30 plasmid encoding L-rhamnose inducible lytic transglycosylase genes, were grown on BHI plates supplemented or not with 1% of L-arabinose and various concentrations of L-rhamnose. To avoid the loss of pHV63(*ycbB*), even in conditions where the production of YcbB is toxic, all plates were supplemented with 20 µg/mL of chloramphenicol. Data are a representative of three biological replicates (*n* = 3).

Previous analyses have shown that endopeptidase MepK is an essential partner of YcbB for the expression of β-lactam resistance mediated by this L,D-transpeptidase, in agreement with preferential cleavage of 3→3 cross-links by this enzyme (25). In the absence of β-lactams, the overproduction of YcbB prevented the growth of a mutant lacking the gene encoding MepK (26). In this study, we determined whether the overproduction of lytic transglycosylases could alleviate the toxicity of YcbB overproduction in a MepK-deficient mutant. Out of the eight lytic transglycosylases, only MltD restored the growth of the Δ*mepK* mutant overproducing YcbB (Fig. 7). The heterologous complementation was not observed for catalytically inactive MltD E^125^A. The overproduction of none of the amidases restored the growth of the Δ*mepK* mutant (Fig. S4). Since MltD is a lipoprotein anchored in the inner leaflet of the outer membrane, alleviation of toxicity might rely on PG turnover at the outer face of the PG layer.

### The toxicity incurred by YcbB overproduction in a Δ*sltY* Δ*mltG* mutant is mitigated by the overproduction of endopeptidase MepK

**Fig 8:**
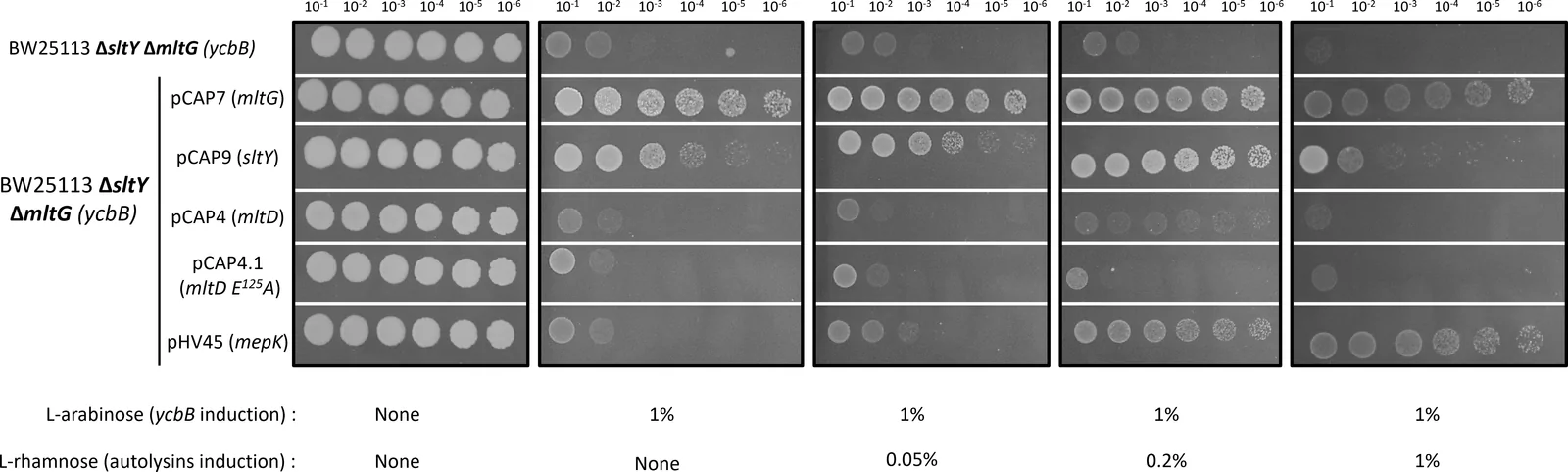
The overproduction of MltD and MepK for the alleviation of YcbB toxicity in a mutant deficient for the SltY and MltG lytic transglycosylases. Derivatives of BW25113 ∆*sltY* ∆*mltG* harboring the pHV63 plasmid allowing L-arabinose-mediated expression of *ycbB* alone, or in combination with derivatives of the pHV30 plasmid encoding the lytic transglycosylases *mltG*, *sltY*, or *mltD*, or the endopeptidase *mepK* were grown on BHI plates supplemented or not with 1% of L-arabinose and various concentrations of L-rhamnose. To avoid the loss of pHV63(*ycbB*), all plates were supplemented with 20 µg/mL of chloramphenicol. Data are a representative of three biological replicates (*n* = 3).

Two lytic transglycosylases are potentially acting from the inner face of the PG layer, SltY, which is a soluble enzyme, and MltG which is anchored to the outer leaflet of the cytoplasmic membrane via an N-terminal hydrophobic segment (Fig. 1; Fig. S1). The overproduction of YcbB was found to be toxic in a mutant deficient in the production of these two lytic transglycosylases (Fig. 8). These observations suggest that the overproduction of YcbB is toxic in the absence of the lytic transglycosylases that are able to act in the space delimited by the outer surface of the cytoplasmic membrane and the inner face of the PG layer. Heterologous complementation assays indicated that the overproduction of MepK was able to restore the growth of the mutant deficient in SltY and MltG production (Fig. 8). In addition, faint growth was observed for the complementation of the Δ*sltY* Δ*mltG* double deletion by the overproduction of MltD. This partial complementation required the lytic transglycosylase activity of MltD because no growth was observed for complementation with the catalytically inactive version of MltD harboring the E^125^A substitution.

## DISCUSSION

Previous analyses have shown that several enzymes involved in the PG metabolism are selectively essential for β-lactam-insensitive PG cross-linking by YcbB (6, 33). This is the case of the D,D-carboxypeptidase PBP5 that generates the tetrapeptide-containing stems specifically used as acyl donors in the transpeptidation reaction catalyzed by YcbB. This is also the case of specific glycosyltransferases, including PBP1b, that polymerize the glycan chains used by YcbB to form a 3→3 cross-linked PG network, as well as specific endopeptidases, including MepK, that cleave 3→3 cross-links. In this study, we extend the repertoire of enzymes conditionally required for the metabolism of 3→3 cross-linked PG with two lytic transglycosylases, MltG and MltD.

**Fig 9:**
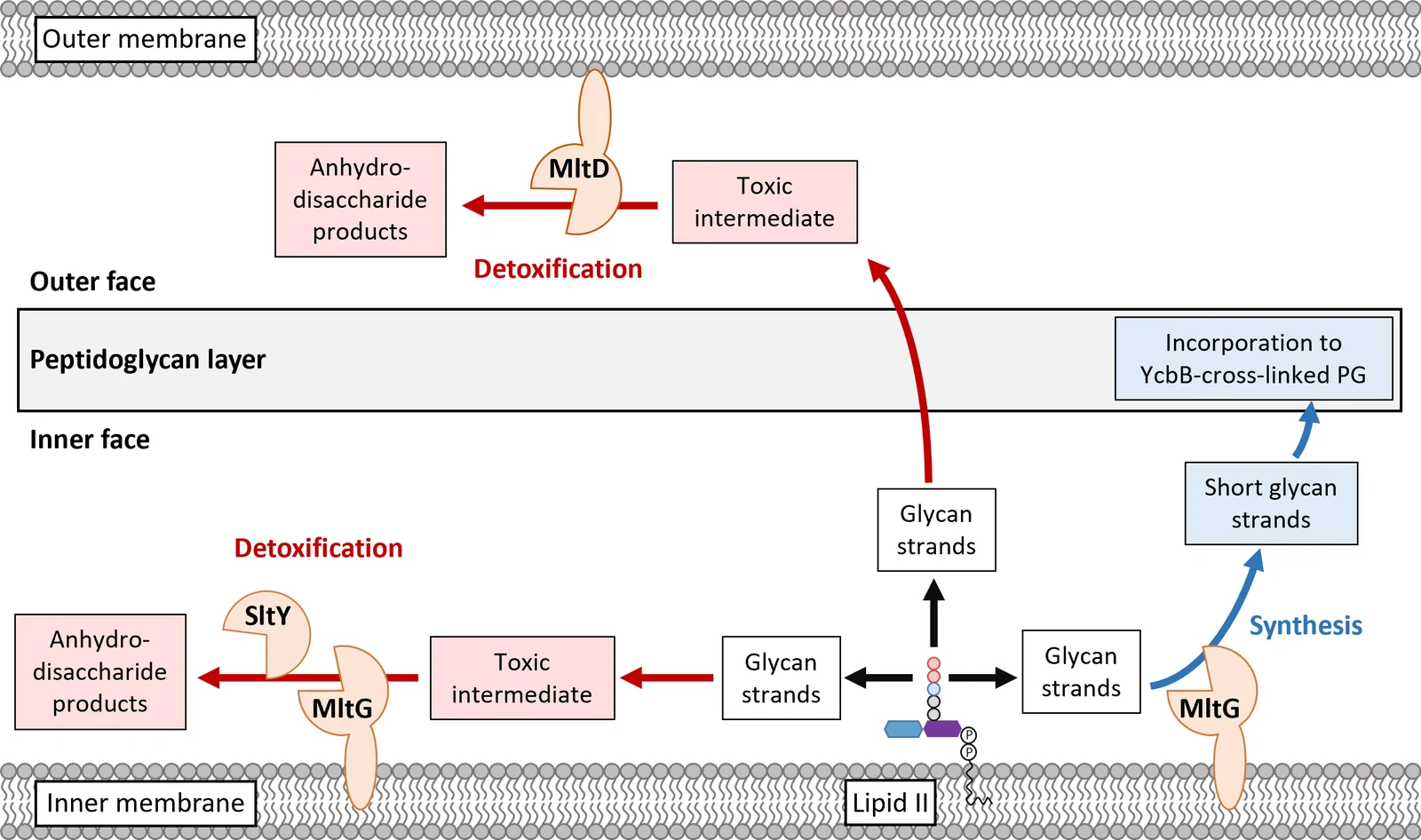
Proposed roles of lytic transglycosylases in L,D-transpeptidase-mediated peptidoglycan metabolism. MltG has a dual role in PG synthesis (blue) and in detoxification of PG intermediates that accumulate at the inner face of the PG layer (red). These intermediates can also be detoxified by SltY. Toxic intermediates accumulating in the outer face of the PG layer can be detoxified by the outer membrane-anchored MltD.

MltG is the only lytic transglycosylase that is tethered to the inner membrane, where glycan chains are polymerized by glycosyltransferases (Fig. 1; Fig. S1). PBP1b interacts with both YcbB and MltG, as shown by pull down experiments and analyses based on two-hybrid systems (16, 34). Thus, nascent glycan chains polymerized by PBP1b should co-localize with MltG indicating that they are potential substrates of this lytic transglycosylase. In agreement, purified MltG cleaves glycan chains polymerized by PBP1b *in vitro* (18). In this study, we showed that purified MltG cleaves glycosidic bonds in purified sacculi, although this enzyme was not active on cross-linked regions of the PG, regardless of the presence of 3→3 or 4→3 cross-links, because muropeptides released from the PG by MltG did not contain any cross-links (Fig. 5). Together, these observations indicate that the role of MltG in the metabolism of 3→3 cross-linked PG depends upon its cleaving activity on nascent glycan chains rather than on mature PG. In order to gain insight into the physiological role of this activity, we investigated two phenotypes stemming from the absence of MltG following the deletion of the corresponding gene. For the first phenotype, we showed that MltG is selectively essential for YcbB-mediated β-lactam resistance (Fig. 2). It was previously established that YcbB is the functional transpeptidase in bacteria grown in the presence of β-lactams due to the inactivation of the D,D-transpeptidase activity of PBPs by the drugs (6). This was experimentally established by detecting the exclusive presence of 3→3 cross-links in the PG (6). As reported in this study (Fig. 5) and in agreement with previous studies (18), MltG has endolytic activity and generates short glycan strands (up to seven disaccharide-peptide units). We propose that these glycan strands are the essential substrates of YcbB (Fig. 9). The latter function is in agreement with the localization of MltG and the participation of this enzyme to a complex comprising PBP1b and YcbB. The second phenotype is the toxicity of the overproduction of YcbB in the absence of β-lactams. In these conditions, YcbB and the PBPs catalyze the formation of 40% and 60% of the cross-links, respectively (5). Under these conditions, the overproduction of YcbB was toxic in a mutant deficient in the production of both MltG and soluble lytic transglycosylase SltY. The latter enzyme is also known to cleave nascent glycan strands, as does MltG (35). However, the activity of SltY is mostly exolytic in contrast to that of MltG which is endolytic (Fig. 5) (13, 18, 35). This difference suggests that SltY is devoid of any biosynthetic role in agreement with the fact that it is dispensable for resistance. Together, these observations indicate that the production of either MltG or SltY is essential to prevent the accumulation of long glycan strands that are toxic in bacteria overproducing YcbB. This uncrowding function has been previously reported for the homolog of MltG in *Vibrio cholerae* (36), SltY in *E. coli* strains grown in the presence of the β-lactam mecillinam (35), and the SltY homolog in *P. aeruginosa* (37, 38). The overproduction of MepK, which cleaves 3→3 cross-links, compensated for the absence of MltG and SltY. Thus, the toxicity resulting from the accumulation of long glycan chains in the absence of SltY and MltG may depend upon their subsequent cross-linking by overproduced YcbB.

The deletion of individual lytic transglycosylase genes identified a second enzyme, MltD, which is essential for full expression of YcbB-mediated β-lactam resistance. Complementation assays indicated that MltD and MltG have non-redundant roles in resistance in spite of the fact that MltG and MltD both displayed an endolytic activity and preferentially cleaved uncross-linked PG. Digestion of sacculi by MltD was strongly impaired by the presence of 3→3 cross-links (Fig. 4). Thus, MltD could not contribute to resistance by specifically cleaving 3→3 cross-linked PG. We, thus, propose that MltD and MltG might both have an uncrowding function. MltD could potentially act on glycan strands protruding through the PG layer. This hypothesis would suggest that the non-redundant role of MltG and MltD could be accounted for by the fact that these enzymes act from the inner and outer faces of the PG layer, in agreement with their anchoring in the inner and outer membrane, respectively (Fig. 9).

## MATERIALS AND METHODS

### Strains, growth conditions, plasmids, and construction of mutants

Strains and plasmids used in this study are detailed in Tables S1 and S2, respectively. All strains used in this study are derivatives of *E. coli* BW25113 (39) except BL21(DE3) used for the production of recombinant proteins (40). All cultures were performed at 37°C in BHI broth under agitation (180 rpm) or on BHI agar plates. The NEBuilder HiFi DNA assembly (New England Biolabs) method was used for the construction of plasmids for this study. The deletion of individual genes was performed by P1-mediated transduction of the Km^R^ cassette of selected mutants from the Keio collection (39). The Km^R^ gene is bordered by two FRT loci, allowing the removal of the cassette by the FLP recombinase whose gene is encoded by plasmid pCP20. The removal of the cassette was performed to allow serial gene deletions (41). To express β-lactam resistance, strains were transformed with plasmids pKT2, allowing IPTG-mediated expression of *ycbB*, and pKT8, allowing L-arabinose-mediated expression of *relA’*. Because *relA’* is co-dominant with the chromosomal *relA* allele, all experiments were carried out in ∆*relA* backgrounds, as reported by Voedts et al. (26).

### Plating efficiency assays

Bacteria were grown to late growth phase to an optical density at 600 nm (OD_600_) greater than 1.0 under agitation (180 rpm), in BHI broth supplemented with the corresponding antibiotic, 10 µg/mL tetracycline, 20 µg/mL chloramphenicol, or 25 µg/mL Zeocine, to counter-select loss of pHV6, pHV7, or pHV30 derivatives, respectively. The OD was adjusted to 1.0, and serial dilutions (10^−1^ to 10^−6^) were performed in BHI broth. Five microliters of each dilution was deposited on BHI agar plates supplemented with the inducers and drugs as indicated in the figure legends. Plates without β-lactams were incubated for 16 h at 37°C, and plates supplemented with ceftriaxone were incubated for 24 h at 37°C.

### Extraction of peptidoglycan

For the preparation of 3→3 cross-linked PG, *E. coli* BW25113 M1.5 pJEH12(*ycbB*) was grown at 37°C to an OD_600 nm_ of 1.5, in 1 L of BHI broth supplemented with 50 µM IPTG (for induction of the *ycbB* gene) and 8 µg/mL ceftriaxone (to inactivate the D,D-transpeptidases belonging to the PBP family). For 4→3 cross-linked PG, *E. coli* BW25113 Δ6*ldt* was grown in the same conditions in un-supplemented BHI broth. PG was extracted by the hot sodium dodecyl sulfate (SDS) procedure (42). Pellets of the bacterial cultures were re-suspended in 120 mL of 4% SDS and boiled until obtaining a non-viscous mixture. The resulting mixtures were centrifuged (53,000 × *g*, 20 min, 30°C) to pellet PG. Three cycles of washes in 30 mL of room temperature water followed by centrifugation (53,000 × *g*, 20 min, 30°C) were performed to remove SDS. PG was digested overnight by 0.2 mg/mL of pronase (10 mM of Tris-HCl [pH 7.5], 37°C). The digestion product was pelleted by centrifugation (53,000 × *g*, 20 min, 30°C) and washed with 2 mL of water. A second overnight digestion step was performed with 0.1 mg/mL of trypsin (20 mM of NaPO_4_ [pH 8.0], 37°C). The digestion product was washed with 2 mL of water and pelleted. Pellets were stored at −20°C.

### Purification of lytic transglycosylases MltD and MltG

The *mltD* and *mltG* coding sequences truncated of the first 48 and 72 5′ nucleotides, respectively, were amplified by PCR and independently cloned into the pET-TEV vector. The resulting recombinant plasmids encoded fusion proteins comprising an N-terminal 6 × His tag and a TEV protease cleavage site. The deduced gene product comprised of residues MGSSHHHHHHSSGENLYFQG fused to residues 16–452 of MltD or residues 24–340 of MltG. The resulting plasmids were introduced *E. coli* BL21(DE3) by transformation. Bacteria were grown in 500 mL of BHI broth up to an OD_600_ of 0.6 at 37°C with vigorous shaking (180 rpm). Induction was performed with 0.5 M of IPTG, and incubation was continued for 2 h in the same conditions. Bacteria were collected by centrifugation, re-suspended in buffer A (25 mM Tris-HCl pH 7.5, 300 mM NaCl), and lysed by sonication. The fusion proteins were purified from clarified lysates in buffer A by Nickel-affinity chromatography (Ni-Sepharose 6 Fast Flow beads; GE Healthcare) and size exclusion chromatography (HiLoad 26/600 Superdex 75 pg; Cytiva). An additional Nickel-affinity chromatography step was performed under denaturation conditions (buffer A containing 8 M urea). Flash renaturation was performed with buffer A on the proteins bound to the resin. Proteins were stored at −20°C in buffer A.

### Preparation and purification of peptidoglycan fragments

The 4→3 and 3→3 cross-linked PG preparations were digested with MltD or MltG at various concentrations (1.6, 0.8, 0.4, and 0.2 mg/mL) for 4 h at 37°C in 50 mM Tris-HCl (pH 7.5). The PG preparations were also digested overnight at 37°C with 1 mg/mL lysozyme in the same buffer, followed by the reduction of MurNAc into muramitol with sodium borohydride (NaBH_4_). Soluble muropeptides were recovered by centrifugation (16,000 × *g* for 10 min at 20°C) and separated by *rp*HPLC in a C18 column (Hypersil GOLD aQ; 250 × 4.6 mm; 3 µm, Thermoscientific) at a flow rate of 1 mL/min with a linear gradient (0% to 20%) applied between 10 and 60 min (buffer 1, TFA 0.1%; buffer 2, acetonitrile 99.9% TFA 0.1%). Absorbance was monitored at 205 nm, and 1-mL fractions were collected and lyophilized. The sum of the *rp*HPLC peak areas obtained by digestion of the PG preparations with MltD, MltG, and lysozyme was determined by the integration of the chromatograms (Chromeleon v7.2.10 ES, Thermo Scientific). The ratios (%) of the sum of the peak areas (MltG-to-lysozyme and MltD-to-lysozyme) were calculated to provide estimates of the extent of digestion of PG by the lytic transglycosylases.

### Mass spectrometry

Muropeptides collected by *rp*HPLC were lyophilized and solubilized in 20 µL of water. A 5-μL aliquot of the fractions was injected into the mass spectrometer (Maxis II ETD, Bruker, France) at a flow rate of 0.1 mL/min (50% acetonitrile, 50% water, 0.1% formic acid; vol/vol), operated in positive mode (Analytical Platform of the Muséum National d’Histoire Naturelle, Paris, France). A capillary voltage of 3,500 V and a 300–1,850 *m*/*z* scan range at a speed of 2 Hz was used, as described in Atze et al*.* (43). Raw data were analyzed using the Compass DataAnalysis software (Bruker, v.4.3).

### Reproducibility

All plating efficiency assays displayed are representative of at least three biological repeats (*n* ≥ 3). For the determination of the cleavage specificities of MltD and MltG, enzymatic digestions of purified sacculi were performed three independent times. The *rp*HPLC profiles shown are representative of three independent digestions in each condition. The full data set can be found in Tables S3 and S4. No data were excluded from the experiments presented in this study.

## Supplementary Material

Reviewer comments

## Data Availability

Raw mass spectrometry data-sets are available in the Zenodo database (https://zenodo.org/records/20183745). The raw data used to generate the figures are provided in Tables S3 and S4.

## SUPPLEMENTAL MATERIAL

Supplemental figures spectrum.01017-26-s0001.docx Fig. S1 to S4.

Tables S1 and S2 spectrum.01017-26-s0002.docx Characteristics and origins of the *E. coli* strains and plasmids used in this study.

Table S3 spectrum.01017-26-s0003.xlsx Source data for Fig. 4.

Table S4 spectrum.01017-26-s0004.xlsx Source data for Fig. 5.

OPEN PEER REVIEW reviewer-comments.pdf An accounting of the reviewer comments and feedback.

ASM does not own the copyrights to Supplemental Material that may be linked to, or accessed through, an article. The authors have granted ASM a non-exclusive, world-wide license to publish the Supplemental Material files. Please contact the corresponding author directly for reuse.
