# Supplementary material for: Essential roles of lytic transglycosylases in l,d-transpeptidase-mediated peptidoglycan cross-linking in Escherichia coli
Source: Microbiol Spectr. 2026 Sep 21;14(11):e01017-26. doi: 10.1128/spectrum.01017-26 (PMC13729221; doi:10.1128/spectrum.01017-26)
Supplement: Supplementary file 1 [file reviewer-comments.pdf]

AMERICAN SOCIETY FOR MICROBIOLOGY | Microbiology Spectrum

Essential roles of lytic transglycosylases in L,D-transpeptidase-mediated peptidoglycan cross-linking in *Escherichia coli*

Constantin Anoyatis-Pelé, Mathilde Benadjaoud, Michel Arthur, and Jean-Emmanuel Hugonnet

Corresponding Author(s): Michel Arthur, INSERM

Review Timeline:

Submission Date:	March 24, 2026
Editorial Decision:	April 13, 2026
Revision Received:	May 19, 2026
Accepted:	May 21, 2026

Editor: Beiyan Nan

Reviewer(s): Disclosure of reviewer identity is with reference to reviewer comments included in decision letter(s). The following individuals involved in review of your submission have agreed to reveal their identity: Tobias Dörr (Reviewer #1)

Transaction Report:

(Note: With the exception of the correction of typographical or spelling errors that could be a source of ambiguity, letters and reports are not edited. The original formatting of letters and referee reports may not be reflected in this compilation.)

DOI: https://doi.org/10.1128/spectrum.01017-26

Re: Spectrum01017-26 (**Essential roles of lytic transglycosylases in L,D-transpeptidase-mediated peptidoglycan cross-linking in *Escherichia coli***)

Dear Dr. Michel Arthur:

Thank you for the privilege of reviewing your work. Below you will find my comments, instructions from the Spectrum editorial office, and the reviewer comments.

Please return the manuscript within 60 days; if you cannot complete the modification within this time period, please contact me. If you do not wish to modify the manuscript and prefer to submit it to another journal, notify me immediately so that the manuscript may be formally withdrawn from consideration by Spectrum.

Revision Guidelines
To submit your modified manuscript, log into the submission site at https://spectrum.msubmit.net/cgi-bin/main.plex. Go to Author Tasks and click the appropriate manuscript title to begin. The information you entered when you first submitted the paper will be displayed; update this as necessary. Note the following requirements:

• Upload point-by-point responses to the issues raised by the reviewers in a file named "Response to Reviewers," NOT in your cover letter.
• Upload a compare copy of the manuscript (without figures) as a "Marked-Up Manuscript" file.
• Upload a clean .DOC/.DOCX version of the revised manuscript and remove the previous version.
• Each figure must be uploaded as a separate, editable, high-resolution file (TIFF or EPS preferred), and any multipanel figures must be assembled into one file.
• Any supplemental material intended for posting by ASM should be uploaded with their legends separate from the main manuscript. You can combine all supplemental material into one file (preferred) or split it into a maximum of 10 files with all associated legends included.

For complete guidelines on revision requirements, see our Submission and Review Process webpage. Submission of a paper that does not conform to guidelines may delay acceptance of your manuscript.

Data availability: ASM policy requires that data be available to the public upon online posting of the article, so please verify all links to sequence records, if present, and make sure that each number retrieves the full record of the data. If a new accession number is not linked or a link is broken, provide Spectrum production staff with the correct URL for the record. If the accession numbers for new data are not publicly accessible before the expected online posting of the article, publication may be delayed; please contact production staff (Spectrum@asmusa.org) immediately with the expected release date.

Publication Fees: For information on publication fees and which article types are subject to charges, visit our website. If your manuscript is accepted for publication and any fees apply, you will be contacted separately about payment during the production process; please follow the instructions in that e-mail. Arrangements for payment must be made before your article is published.

ASM Membership: Corresponding authors may join or renew ASM membership to obtain discounts on publication fees. Need to upgrade your membership level? Please contact Customer Service at Service@asmusa.org.

The ASM Journals program strives for constant improvement in our submission and publication process. Please tell us how we can improve your experience by taking this quick Author Survey.

Thank you for submitting your paper to Spectrum.

Sincerely,
Beiyan Nan
Editor
Microbiology Spectrum

Reviewer #1 (Comments for the Author):

In this manuscript, Anoyatis-Pele et al. dissect the involvement/requirement of lytic transglycosylases in growth and beta lactam resistance of a strain relying on 3,3 crosslinks for PG synthesis. The main conclusions are that MltG is a major provider of the substrate for 3,3 crosslinks by YcbB, but also detoxifies detrimental bioproducts of PG synthesis, while MltD is a major contributor to the latter function only. Overall, the conclusions are well-supported by the data and the study is straight-forward

and well-designed.

Minor comments
- Fig. 2B/3B. I find it interesting that there seems to be a sweet spot for mltG and MltD expression in panel B (i.e. rescue is most pronounced at low inducer concentration). If MltG and MltD only provided substrate and detoxified toxic byproducts, one would assume that more is always better. Or is MltG and MltD overexpression at those levels just generally toxic (i.e., even in WT)?
- Fig. 6 - I would be more conservative and call this "partial" restoration of resistance (line 273). The phenotype is subtle, and only observed at highest inducer concentration.
- Line 297/298. To really draw this conclusion you might consider swapping experiments, where you forcefully localize MltD to the inner membrane (and MltG to the outer). Alternatively, please weaken this statement ("since MltD localizes to the outer membrane, it is likely that alleviation of toxicity relies on PG turnover at the outer face of the PG layer" or something like that). I recommend the latter, since the swapping experiments might cause some toxicity.
Very minor comments
- Line 73 either "glycosyltransferases that polymerize..." or "glycosyltransferases, which polymerize". Same in the second part of the sentence.
- Line 82 "non-essential"
- Line 118, comprise is the wrong word. Maybe "Autolysins contain"?
- Line 345 - remove "physically"- if I am not mistaken, that paper used BACTH but explicitly stated that they cannot rule out indirect interactions (it is possible that MltG and PBP1B just happen to localize to the same substrate).

Reviewer #2 (Comments for the Author):

The manuscript titled "Essential roles of lytic transglycosylases in L,D-transpeptidase-mediated peptidoglycan cross-linking in Escherichia coli" reports that MltD and MltG are essential for the survival of E. coli overproducing LdtD during β-lactam antibiotic exposure. The authors propose that this survival depends on the removal of un-crosslinked glycan strands in the periplasm by MltD and MltG, as their accumulation is toxic to the cell. In addition, the study suggests a distinct role for MltG in generating short glycan substrates that are utilized by LdtD for cross-linking. Furthermore, it is reported that overexpression of MepK can alleviate the growth defect associated with MltG deletion. The evidence provided to support the essentiality of MltD and MltG under the aforementioned conditions is convincing. However, the proposed additional role of MltG in generating substrates for LdtD-mediated cross-linking is not sufficiently supported. Previous studies have established that MltD and MltG help alleviate periplasmic crowding by removing uncrosslinked glycan strands, and the current study reinforces and extends these findings.

Following are my comments:

1. My major concern is that almost all the important conclusions of this manuscript are based on viability assays done on agar plates. I suggest that in some important cases, these have to be established using either growth curves (in liquid cultures) or by microscopy (These need to be done for the results shown in Figs. 2, 6 and 8).

2. Line 197 onwards: This section highlights that MltD acts on uncrosslinked glycans which has already been shown in E. coli by Kaul et al., 2024 and this reference should be cited in this section. Similarly, previous studies by Yunck et al., 2016 and Sassine et al., 2021 showed the endolytic activity of MltG on nascent glycan strands which must be mentioned here as well. The authors have reconfirmed previous observations in this section, with additional insights into 4-3 and 3-3 crosslinked PG as substrates.

3. The statement in Line 227 that MltD prefers 4-3 crosslinked PG raises a potential inconsistency. If MltD indeed shows limited activity toward 3-3 crosslinked PG, it is unclear why deletion of mltD is lethal in cells producing predominantly 3-3 crosslinks (as shown in Fig. 2A). The authors should clarify this apparent discrepancy and provide a mechanistic explanation.

4. Line 208-210. The conclusion that MltD and MltG preferentially act on uncrosslinked peptidoglycan (PG) is based on a comparative analysis of their enzymatic activity with that of lysozyme. However, this comparison may not be appropriate. The enzyme preparations of MltD and MltG could differ in terms of stability, purity, and the presence of contaminating proteins, all of which may influence the observed activity. Authors can re-state these results.

5. Line 228-230. This statement can be rephrased to have clarity.

6. Line 297-298, 389. The statement made in these lines is not sufficiently supported by experimental evidence. While MltD is reported to localize to the outer membrane, it remains unclear how its activity can be spatially restricted or distinguished between the outer and inner faces of the peptidoglycan (PG) layer within the context of Gram-negative cell envelope architecture.

7. Lines 382-393. The localisation of MltD and MltG does not necessarily explain their redundancy. The idea of MltG and MltD working on PG from different sides is overstated and oversimplified. They have been shown to have very distinct roles in PG

metabolism by others. This section should be toned down.

8. Several typos are there in the manuscript. They need to be carefully edited.

9. Running title is not appropriate- this manuscript does not show that L,D-transpeptidation requires LTs activity.

10. The modified nomenclature of Ldts is being used now in E. coli field. Suggest using LdtD rather than YcbB in the manuscript to avoid confusion between the researchers.

11. Lines 118: Amidases also have a major role in recycling (in addition to cell-cell separation).

Answer to the reviewers:

Reviewer #1 (Comments for the Author):

In this manuscript, Anoyatis-Pele et al. dissect the involvement/requirement of lytic transglycosylases in growth and beta lactam resistance of a strain relying on 3,3 crosslinks for PG synthesis. The main conclusions are that MltG is a major provider of the substrate for 3,3 crosslinks by YcbB, but also detoxifies detrimental bioproducts of PG synthesis, while MltD is a major contributor to the latter function only. Overall, the conclusions are well-supported by the data and the study is straight-forward and well-designed.

__Answer:__ We thank the reviewer for these positive comments.

Reviewer #1, minor comments

Reviewer #1, comment #1

1. Fig. 2B/3B. I find it interesting that there seems to be a sweet spot for mltG and MltD expression in panel B (i.e. rescue is most pronounced at low inducer concentration). If MltG and MltD only provided substrate and detoxified toxic byproducts, one would assume that more is always better. Or is MltG and MltD overexpression at those levels just generally toxic (i.e., even in WT)?

__Answer:__ To investigate the putative toxicity of MltD and MltG overproduction, we expressed *mltD* and *mltG* in an *E. coli* host strain harboring the full set of lytic transglycosylase-encoding genes ("wild-type" instead of the Δ*mltG* or Δ*mltD* background). The expression of *mltD* and *mltG* was obtained using the same plasmids as in the experiments depicted in Fig. 2B and 3B of the submitted manuscript. The results (figure below) showed that the plating efficiency in the presence of ceftriaxone was reduced in response to induction of *mltD* and *mltG*. As suggested by the reviewer, this observation suggests that high-level production MltG and MltD is toxic. This effect is only observed in the presence of ceftriaxone probably because subinhibitory concentrations of this drug compromised the functions of the cell envelope, as discussed in one of our previous publications (PMID: 36575173). We believe that this observation is somewhat expected, and, for the sake of simplicity, we prefer not to incorporate the data in the revised version of our manuscript.

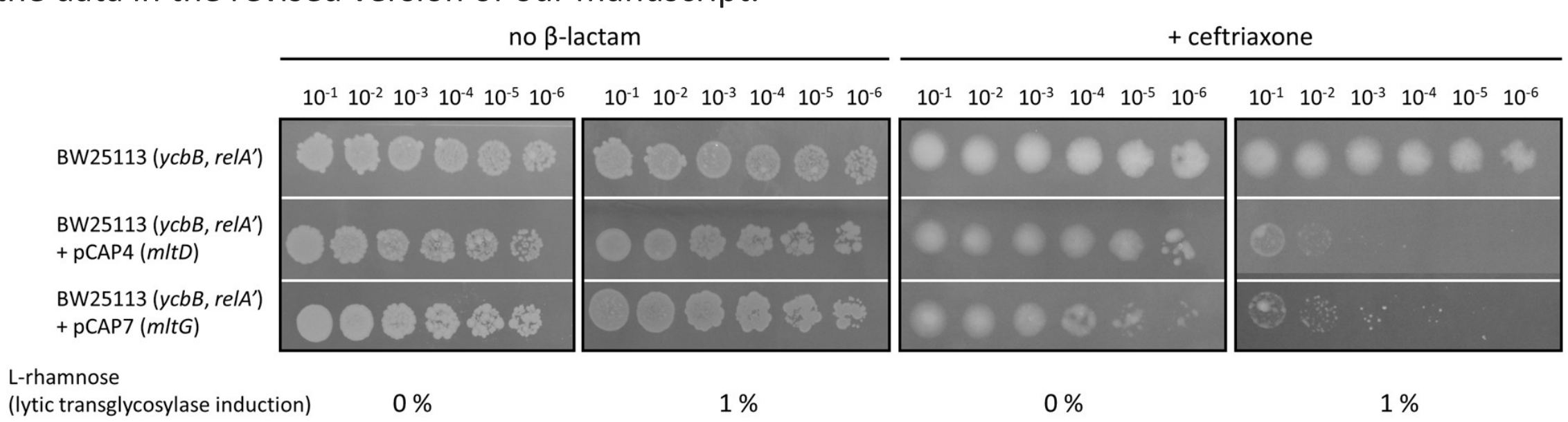

Reviewer #1, comment #2

2. Fig. 6 - I would be more conservative and call this "partial" restoration of resistance (line 273). The phenotype is subtle, and only observed at highest inducer concentration.

__Answer:__ As requested, we have added the term "partial" to the corresponding line. The revised sentence now reads: "This partial complementation was dependent upon the endopeptidase activity of MepK […]" (line 279 of the revised manuscript)

Reviewer #1, comment #3

3. Line 297/298. To really draw this conclusion you might consider swapping experiments, where you forcefully localize MltD to the inner membrane (and MltG to the outer). Alternatively, please weaken this statement ("since MltD localizes to the outer membrane, it is likely that alleviation of toxicity relies on PG turnover at the outer face of the PG layer" or something like that). I recommend the latter, since the swapping experiments might cause some toxicity.

Answer: As requested, we have softened the statement originally appearing in lines 297-298. The new sentence reads: "Since MltD is a lipoprotein anchored in the inner leaflet of the outer membrane, alleviation of toxicity might rely on PG turnover at the outer face of the PG layer" (lines 302-303 of the revised manuscript).

Reviewer #1, additional comments

Very minor comments

- Line 73 either "glycosyltransferases that polymerize..." or "glycosyltransferases, which polymerize". Same in the second part of the sentence.

- Line 82 "non-essential"

- Line 118, comprise is the wrong word. Maybe "Autolysins contain"?

- Line 345 - remove "physically"- if I am not mistaken, that paper used BACTH but explicitly stated that they cannot rule out indirect interactions (it is possible that MltG and PBP1B just happen to localize to the same substrate).

Answer: We have corrected all four indicated lines.

Reviewer #2 (Comments for the Author):

The manuscript titled "Essential roles of lytic transglycosylases in L,D-transpeptidase-mediated peptidoglycan cross-linking in Escherichia coli" reports that MltD and MltG are essential for the survival of E. coli overproducing LdtD during β-lactam antibiotic exposure. The authors propose that this survival depends on the removal of un-crosslinked glycan strands in the periplasm by MltD and MltG, as their accumulation is toxic to the cell. In addition, the study suggests a distinct role for MltG in generating short glycan substrates that are utilized by LdtD for cross-linking. Furthermore, it is reported that overexpression of MepK can alleviate the growth defect associated with MltG deletion. The evidence provided to support the essentiality of MltD and MltG under the aforementioned conditions is convincing. However, the proposed additional role of MltG in generating substrates for LdtD-mediated cross-linking is not sufficiently supported. Previous studies have established that MltD and MltG help alleviate periplasmic crowding by removing uncrosslinked glycan strands, and the current study reinforces and extends these findings.

Answer: We thank the reviewer for his thorough evaluation of our work.

Reviewer #2, comment #1

1. My major concern is that almost all the important conclusions of this manuscript are based on viability assays done on agar plates. I suggest that in some important cases, these have to be established using either growth curves (in liquid cultures) or by microscopy (These need to be done for the results shown in Figs. 2, 6 and 8).

Answer: We chose to analyze the β-lactam resistance phenotypes by using plating efficiency assays because previous studies showed that YcbB-producing *E. coli* strains do not express β-lactam resistance in liquid medium (ref 26). Thus, the requested experiments cannot be performed for the

strains analyzed in Fig. 2 and 6. In addition, we would like to stress that the plating efficiency assays provided information on the portion of the inoculum that grows in the presence of β-lactams, which we believe is a clearer readout than the growth rate and maximum yield provided by an analysis of growth in liquid medium.

Nonetheless, to provide the requested additional analysis, we have compared growth in liquid medium of the strains depicted in Fig. 8 (figure below). This analysis established, as expected, that overproduction of either MepK or StlY mitigates the toxicity incurred by the overproduction of YcbB in the BW25113 Δ*sltY* Δ*mltG* pHV63(*ycbB*) background.

It was not possible to determine whether production of MltD improved growth of strain BW25113 Δ*sltY* Δ*mltG* pHV63(*ycbB*) in condition of induced expression of the *ycbB* gene. This was due to the intrinsic toxicity of MltD production in liquid medium, a toxicity that was also observed for the catalytically inactive MltD E^{125}A. Thus, monitoring growth in liquid medium cannot be used to highlight the fitness benefit of the production of MltD in the context of *ycbB* induction.

For MltG, production of the lytic transglycosylase partially mitigated the toxicity incurred by overproduction of YcbB in the BW25113 Δ*sltY* Δ*mltG* pHV63(*ycbB*) background, similar to what was observed for SltY (above), except that induction of *mltG* was in itself toxic in the absence of *ycbB* induction. Thus, analysis in liquid medium is unsuitable for comparing all autolysins in our complementation study due to differential toxicity related to their production. Likewise, microscopy analysis is not suitable for this purpose since production of the autolysins and drug exposure are known to result is various morphology defects. For these reasons, we prefer not including the results displayed below in the revised version of the manuscript.

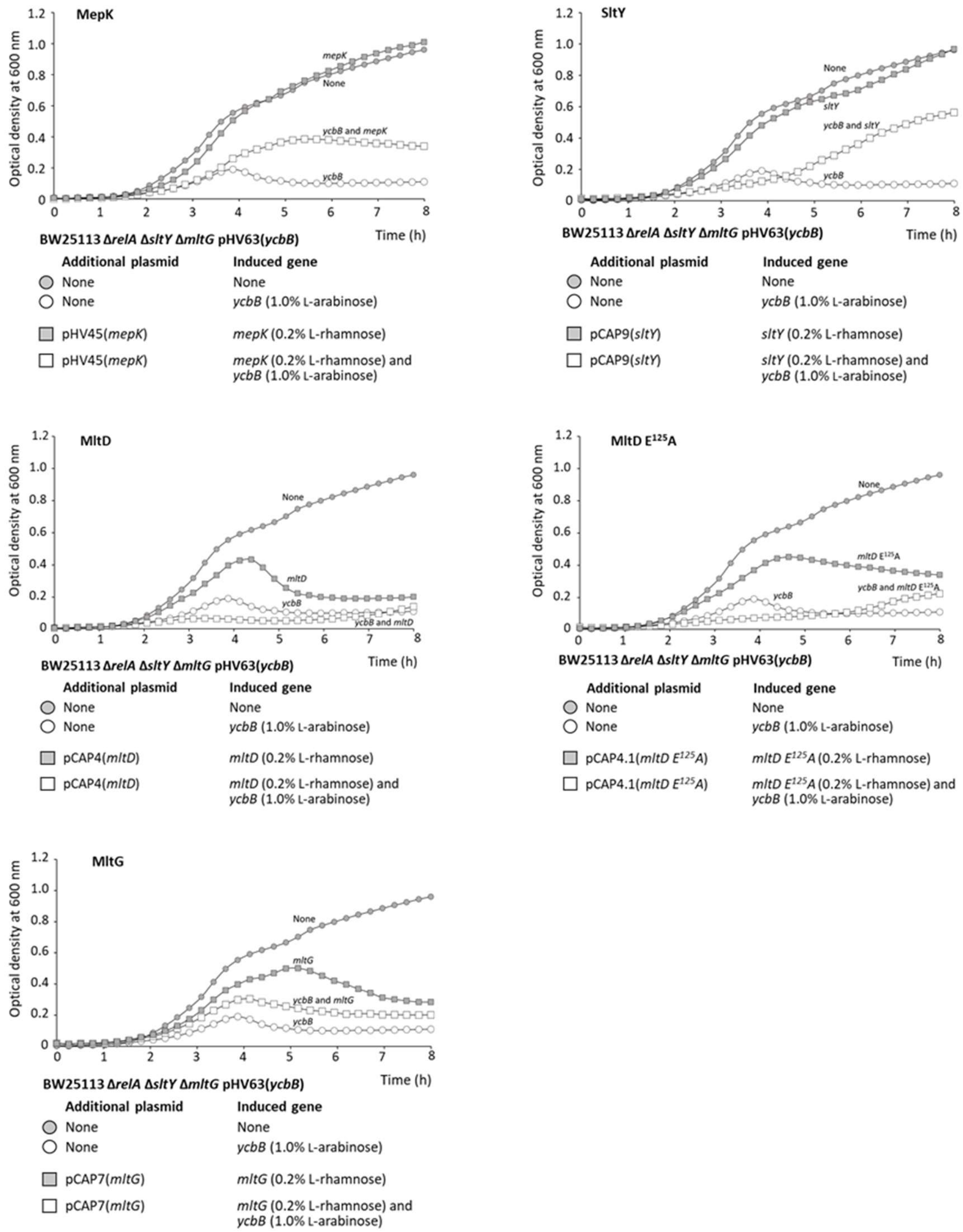

Figure legend: Monitoring growth in liquid medium. For each tested strain, a fresh colony was inoculated in 10 ml BHI broth and incubated overnight at 37 °C. The media contained 20 µg/ml chloramphenicol and 25 µg/ml zeocine to counteract loss of the plasmids expressing *ycbB* and the autolysine genes, respectively. The next day 200 µl of BHI broth in 96 well were inoculated with the overnight culture to obtain an optical density at 600 nm of 0.03. The media also contained zeocine and chloramphenicol at the concentrations listed above. Induction of autolysin genes and of *ycbB* was performed with 0.2% L-rhamnose and 1.0% L-arabinose, respectively. OD_{600} was continuously monitored with a plate reader (Tecan Infinite 200 pro) at 37 °C with agitation. Data are the average of two technical replicates obtained for each one of three biological replicates.

Reviewer #2, comment #2

2. Line 197 onwards: This section highlights that MltD acts on uncrosslinked glycans which has already been shown in E. coli by Kaul et al., 2024 and this reference should be cited in this section. Similarly, previous studies by Yunck et al., 2016 and Sassine et al., 2021 showed the endolytic activity of MltG on nascent glycan strands which must be mentioned here as well. The authors have reconfirmed previous observations in this section, with additional insights into 4-3 and 3-3 crosslinked PG as substrates.

Answer: As requested by the reviewer, we have added the reference to the study published by Kaul *et al*. (2024), which also reports the preference of MltD for uncross-linked substrates.

Lines 218-219: Thus, MltD preferentially cleaves uncross-linked regions of the PG, as previously reported (13, 31).

To emphasize previous findings on the endolytic activity of MltG, we have added a reference describing the activity of MltG on nascent glycan strands. We also added the *Yunck et al. 2016* reference in this sentence, which previously only referenced the work by *Sassine et al., 2021*. The revised sentence lines 202-204 reads: "A similar cleavage specificity was previously reported for MltG, which was shown to act on nascent glycan strands (16,18)"

Reviewer #2, comment #3

3. The statement in Line 227 that MltD prefers 4-3 crosslinked PG raises a potential inconsistency. If MltD indeed shows limited activity toward 3-3 crosslinked PG, it is unclear why deletion of mltD is lethal in cells producing predominantly 3-3 crosslinks (as shown in Fig. 2A). The authors should clarify this apparent discrepancy and provide a mechanistic explanation.

Answer: The reviewer is correct in stating that MltD shows very low activity toward 3→3 crosslinked PG. Nonetheless, MltD is essential in cells predominantly producing 3→3 crosslinked PG because this lytic transglycosylase acts prior to the cross-linking of glycan chains by YcbB. We apologize if the first version was unclear and rephrase the corresponding section.

First version

The cleavage preference of MltD and MltG for uncross-linked PG indicates that these lytic transglycosylases act either on glycan chains that are not yet cross-linked by YcbB, or on previously cross-linked substrates whose cross-links were cleaved by endopeptidases.

Revised version (lines 228-233 of the revised manuscript)

The cleavage preference of MltD and MltG for uncross-linked PG indicates that these lytic transglycosylases act on glycan chains prior to their cross-linking by transpeptidases. This could account for the fact that MltD is essential in cells predominantly producing 3→3 crosslinked PG although this lytic transglycosylase does not display a preference for PG containing this type of cross-links. The specificity of MltD for uncross-linked PG indicates that this enzyme could also act in combination with endopeptidases.

Reviewer #2, comment #4

4. Line 208-210. The conclusion that MltD and MltG preferentially act on uncrosslinked peptidoglycan (PG) is based on a comparative analysis of their enzymatic activity with that of lysozyme. However, this comparison may not be appropriate. The enzyme preparations of MltD and MltG could differ in terms of stability, purity, and the presence of contaminating proteins, all of which may influence the observed activity. Authors can re-state these results.

Answer: We would like to emphasize that one of the main conclusion of our study (last paragraph of the discussion) is that the non-redundant roles of MltG and MltD in PG metabolism stems from the localization of the enzymes, and not form a difference in the substrate specificity of these enzymes, as detected by experiments based on *in vitro* digestion of sacculi (Fig. 5). Nonetheless, we have added a sentence to mention potential limitations of our study, as requested by the reviewer (Line 233-236):

However, it should be acknowledged that differences in the stability and purity of our preparations of MltG and MltD might account for minor differences detected in the substrate specificity of these enzymes.

Reviewer #2, comment #5
5. Line 228-230. This statement can be rephrased to have clarity.

Answer: This comment was addressed in our answer to comment #3

Reviewer #2, comment #6
6. Line 297-298, 389. The statement made in these lines is not sufficiently supported by experimental evidence. While MltD is reported to localize to the outer membrane, it remains unclear how its activity can be spatially restricted or distinguished between the outer and inner faces of the peptidoglycan (PG) layer within the context of Gram-negative cell envelope architecture.

Answer: While we agree that no direct experimental evidence is provided for the fact that MltD and MltG could act on the outer and inner face of the peptidoglycan layer, respectively, this hypothesis would be consistent with the fact that they have non-redundant essential roles and their reported localization. As requested, and in agreement with a similar comment (#3) from reviewer #1, we have softened the statement originally appearing Lines 301-303. The new sentence reads: "Since MltD is a lipoprotein anchored in the inner leaflet of the outer membrane, alleviation of toxicity might rely on PG turnover at the outer face of the PG layer".
(see answer to comment #7 for the comment on line 389).

Reviewer #2, comment #7
7. Lines 382-393. The localisation of MltD and MltG does not necessarily explain their redundancy. The idea of MltG and MltD working on PG from different sides is overstated and oversimplified. They have been shown to have very distinct roles in PG metabolism by others. This section should be toned down.

Answer: As requested, we have toned down the corresponding sequence. The revised version now reads: "We thus propose that MltD and MltG might both have an uncrowding function. MltD could potentially act on glycan strands protruding through the PG layer. This hypothesis would suggest that the non-redundant role of MltG and MltD could be accounted for by the fact that these enzymes act from the inner and outer faces of the PG layer, in agreement with their anchoring in the inner and outer membrane, respectively (Fig. 9)." (lines 392-396)

Reviewer #2, comment #8
8. Several typos are there in the manuscript. They need to be carefully edited.

Answer: Remaining typos have been corrected.

Reviewer #2, comment #9
9. Running title is not appropriate- this manuscript does not show that L,D-transpeptidation requires LTs activity.

Answer: As requested, we revised the running title:
LTs involved in the metabolism of 3→3 cross-linked PG (revised version)
Instead of:
L,D-transpeptidation requires lytic transglycosylases (first version)

Reviewer #2, comment #10

10. The modified nomenclature of Ldts is being used now in E. coli field. Suggest using LdtD rather than YcbB in the manuscript to avoid confusion between the researchers.

Answer: We have added both names in the manuscript to avoid any confusion "aka LdtD" in the first occurrence in the abstract, importance, and introduction paragraphs.

Reviewer #2, comment #11

11. Lines 118: Amidases also have a major role in recycling (in addition to cell-cell separation).

Answer: We have added a sentence and one reference to mention the role of amidases in PG recycling.

Lines 120-121: Amidases are also involved in PG recycling (30).

Re: Spectrum01017-26R1 (**Essential roles of lytic transglycosylases in L,D-transpeptidase-mediated peptidoglycan cross-linking in *Escherichia coli***)

Dear Dr. Michel Arthur:

Your manuscript has been accepted, and I am forwarding it to the ASM production staff for publication. Your paper will first be checked to make sure all elements meet the technical requirements. ASM staff will contact you if anything needs to be revised before copyediting and production can begin. Otherwise, you will be notified when your proofs are ready to be viewed.

Data Availability: ASM policy requires that data be available to the public upon online posting of the article, so please verify all links to sequence records, if present, and make sure that each number retrieves the full record of the data. If a new accession number is not linked or a link is broken, provide production staff with the correct URL for the record. If the accession numbers for new data are not publicly accessible before the expected online posting of the article, publication may be delayed; please contact ASM production staff immediately with the expected release date.

Publication Fees: For information on publication fees and which article types have charges, please visit our website. We have partnered with Copyright Clearance Center (CCC) to collect author charges. If fees apply to your paper, you will receive a message from no-reply@copyright.com with further instructions. For questions related to paying charges through RightsLink, please contact CCC at ASM_Support@copyright.com or toll free at +1-877-622-5543. CCC makes every attempt to respond to all emails within 24 hours.

ASM Membership: Corresponding authors may join or renew ASM membership to obtain discounts on publication fees. Need to upgrade your membership level? Please contact Customer Service at Service@asmusa.org.

Embargo Policy: A press release may be issued as soon as the manuscript is posted on the Spectrum Latest Articles webpage. The corresponding author will receive an email with the subject line "ASM Journals Author Services Notification" when the article is available online.

The ASM Journals program strives for constant improvement in our submission and publication process. Please tell us how we can improve your experience by taking this quick Author Survey.

Thank you for submitting your paper to Spectrum.

Sincerely,
Beiyan Nan
Editor
Microbiology Spectrum
