# Supplementary material for: Essential roles of lytic transglycosylases in l,d-transpeptidase-mediated peptidoglycan cross-linking in Escherichia coli
Source: Microbiol Spectr. 2026 Sep 21;14(11):e01017-26. doi: 10.1128/spectrum.01017-26 (PMC13729221; doi:10.1128/spectrum.01017-26)
Supplement: Supplementary file 2 [file spectrum.01017-26-s0001.docx]

**Supplementary Figure S1. Domain composition of lytic transglycosylases and endopeptidases.** **(A)** Domain composition of *E. coli* lytic transglycosylases. Enzymatic activity was detected for all enzymes (13, 16) except for RlpA, which is closely related (45% identity) to *Pseudomonas aeruginosa* RlpA (19). *P. aeruginosa* RlpA is catalytically active whereas *E*. coli RlpA is not, presumably due to substitution of the catalytic Asp by a Ser residue. Ser at position 147 is present in RlpA of the BW25113 strain used in our study, as well as in other *E*. *coli* K12-derivative strains. MltC, MltE, MltD, MltA, MltB, and RlpA are secreted by the Sec/SPII pathway and harbor Cys residues (pink) undergoing palmitoylation for protein anchoring to the inner leaflet of the outer membrane. SltY is a soluble enzyme addressed to the periplasm by the Sec/SPI pathway. MltG is located in the periplasm and is anchored to the cytoplasmic membrane. MltF is bound to the outer membrane (46) but is devoid of both any recognizable signal peptide and putative palmitoylated cysteine residue. **(B)** Domain composition of *E. coli* endopeptidases. Enzymatic activity was reported for all eight endopeptidases. MepH, MepA, MepK, and PBP7 are soluble enzymes addressed to the periplasm by the Sec/SPI pathway. MepS is the only endopeptidase secreted by the Sec/SPII pathway and anchored to the inner leaflet of the outer membrane by a palmitoylated Cys residue (pink). MepM is anchored to the inner membrane via a hydrophobic domain. DacB and AmpH can exist either in soluble form or anchored to the inner membrane. Pfam domains with their assigned name are represented by colored boxes. Predicted catalytic residues are presented in blue. Signal peptide prediction was performed with SignalP 5.0 (47).

**Supplementary Figure S2. Functional complementation of the *mltG* and *mltD* deletions by endopeptidases. (A)** The plating efficiency of BW25113 ∆*mltG* (*ycbB, relA’*) or its derivatives harboring plasmids enabling L-rhamnose-inducible expression of the genes encoding each one of the eight endopeptidases was tested in BHI agar plates supplemented with 40 µM IPTG, (1%) L-arabinose, 8 µg/mL ceftriaxone (+ ceftriaxone) or in the absence of the drug (- β-lactam). The concentration of L-rhamnose (%) for induction of endopeptidase genes varied as indicated at the bottom of the panels. **(B)** The same assay as described in (A) was performed for the BW25113 ∆*mltD* (*ycbB, relA’*) strain. As a control, strains were transformed with the pHV30 vector (empty). Data are representative of three independent experiments (n = 3).

**Supplementary Figure S3. Functional complementation of the *mltG* and *mltD* deletions by amidases. (A)** The plating efficiency of BW25113 ∆*mltD* (*ycbB, relA’*) or its derivatives harboring plasmids enabling L-rhamnose-inducible expression of the genes encoding each one of the three amidases was tested in BHI agar plates supplemented with 40 µM IPTG, (1%) L-arabinose, 8 µg/mL ceftriaxone (+ ceftriaxone) or in the absence of the drug (- β-lactam). The concentration of L-rhamnose (%) for induction of amidases genes varied as indicated at the bottom of the panels. **(B)** The same assay as described in (A) was performed for the BW25113 ∆*mltG* (*ycbB, relA’*) strain. Data are representative of three independent experiments (n = 3).

**Supplementary Figure S4. Functional complementation of the *mepK* deletion by amidases.** Derivatives of BW25113 ∆*mepK* harboring the pHV63 plasmid allowing L-arabinose-mediated expression of *ycbB* alone, or in combination with derivatives of the pHV30 plasmid encoding L-rhamnose inducible amidase genes, were grown on BHI plates supplemented or not with 1% of L-arabinose and various concentrations of L-rhamnose. As a control, BW25113 ∆*mepK* (*ycbB*) strain harboring plasmid pHV45 (*mepK*) was used. To avoid the loss of pHV63(*ycbB*), even in conditions where production of YcbB is toxic, all plates were supplemented with 20 µg/mL of chloramphenicol. Data are a representative of three biological replicates (n = 3).
