# Supplementary material for: Essential roles of lytic transglycosylases in l,d-transpeptidase-mediated peptidoglycan cross-linking in Escherichia coli
Source: Microbiol Spectr. 2026 Sep 21;14(11):e01017-26. doi: 10.1128/spectrum.01017-26 (PMC13729221; doi:10.1128/spectrum.01017-26)
Supplement: Supplementary file 3 [file spectrum.01017-26-s0002.docx]

**Supplementary Table S1. Characteristics and origin of the *E. coli* strains used in this study**

| **Strain** | **Characteristics** | **Origin** |
| --- | --- | --- |
| BW25113 | *E. coli* K12 derivative | (39) |
| BW25113 (*ycbB*; *relA’*) | Derivative of BW25113 harboring plasmids pKT2(*ycbB*) and pKT8(*relA’*)  and a deletion of the chromosomal copy of *relA*. | (6, 26) |
| BW25113 ∆*mltA*, ∆*mltB*, ∆*mltC*, ∆*mltD*, ∆*mltE*,  ∆*mltF*, ∆*mltG*, ∆*rlpA*, or ∆*sltY* (*ycbB*; *relA’*) | Derivatives of BW25113 (*ycbB*; *relA’*), harboring plasmids pKT2(*ycbB*) and pKT8(*relA’*) a deletion of the chromosomal copy of *relA* and either *mltA*, *mltB*,  *mltC*, *mltD*, *mltE*, *mltF*, *mltG*, *rlpA*, or *sltY*. | This study |
| BW25113 ∆*sltY* ∆*mltG* (*ycbB*; *relA’*) | Derivative of BW25113 (*ycbB*; *relA’*), harboring plasmids pKT2(*ycbB*) and  pKT8(*relA’*) and a deletion of the chromosomal copies of *relA*, *sltY*, and *mltG*. | This study |
| BW25113 ∆*mepK* (*ycbB*) | Derivative of BW25113 harboring plasmid pHV63(*ycbB*) and deletions of genes  *relA* and *mepK* | (26) |
| BW25113 M1.5 (*ycbB*) | Ampicillin resistant derivative of BW25113 pJEH12(*ycbB*) harboring a mutation  in the 5’ UTR region of *ileRS* and the insertion of IS*1* in gene *sltY* | (26) |
| BW25113 ∆*6ldt* | Derivative of BW25113 harboring deletions of genes *ycbB*, *ynhG*, *ycfS*, *erfK*, *ybiS*, and *yafK* | (42) |
| BL21(DE3) | Host for protein production | (40) |

**Supplementary Table S2. Characteristics and origin of the plasmids used for this study**

|  | **Plasmid** | **Characteristics** | | | | | **Origin** |
| --- | --- | --- | --- | --- | --- | --- | --- |
| *Vectors* | | | | | | | |
|  | pHV6 | Tet^R^ | P*_trc_* | | *lacI* | CloDF13 | (26) |
|  | pHV7 | Cm^R^ | P*_araBAD_* | | *araC* | p15A | (26) |
|  | pHV30 | Zeo^R^ | P*_rhaBAD_* | | *rhaSR* | pSC101 | (26) |
|  | pET-TEV | Km^R^ | P_T7(_*_lac_*_)_ | | *lacI* | ColE1 | (43) |
| *Recombinant plasmids for ycbB and relA’ expression* | | | | | | | |
|  | pKT2 | pHV6Ω*ycbB* | | IPTG-inducible expression of *ycbB* | | | (6) |
|  | pKT8 | pHV7Ω*relA’* | | L-arabinose-inducible expression of *relA’* | | | (6) |
|  | pHV63 | pHV7Ω*ycbB* | | L-arabinose-inducible expression of *ycbB* | | | (26) |
| *Recombinant plasmids for endopeptidase-encoding genes expression* | | | | | | | |
|  | pHV43; 44; 45; 45.1; 46;  47; 48; 49; and 50 | pHV30Ω*mepA*; *mepH*; *mepK*; *mepK* H^133^A; *mepM*; *mepS*; *dacB*;  *pbpG*; and *ampH* | | | | | (26) |
| *Recombinant plasmids for lytic transglycosylase-encoding genes expression* | | | | | | | |
|  | pCAP1; 2; 3; 4; 4.1; 5; 6;  7; 8; and 9 | pHV30Ω*mltA*; *mltB*; *mltC*; *mltD*; *mltD* E^125^A; *mltE*; *mltF*; *mltG*; *rlpA*;  and *sltY* | | | | | This study |
| *Recombinant plasmids for amidase-encoding genes expression* | | | | | | | |
|  | pCAP17; 18; 19 | pHV30Ω*amiA*; *amiB*; *amiC* | | | | | This study |
| *Recombinant plasmids for protein production* | | | | | | | |
|  | pCAP13 | pET-TEVΩ*mltD*∆1-48 | | | | | This study |
|  | pCAP16 | pET-TEVΩ*mltG*∆1-72 | | | | |  |

All recombinant plasmids were sequenced by Sanger or Nanopore methods (Eurofins Genomics).
